# Extensive Phylogenomic Discordance and the Complex Evolutionary History of the Neotropical Cat Genus *Leopardus*

**DOI:** 10.1093/molbev/msad255

**Published:** 2023-11-21

**Authors:** Jonas Lescroart, Alejandra Bonilla-Sánchez, Constanza Napolitano, Diana L Buitrago-Torres, Héctor E Ramírez-Chaves, Paola Pulido-Santacruz, William J Murphy, Hannes Svardal, Eduardo Eizirik

**Affiliations:** Department of Biology, University of Antwerp, Antwerp, Belgium; School of Health and Life Sciences, Pontifical Catholic University of Rio Grande do Sul, Porto Alegre, Brazil; School of Health and Life Sciences, Pontifical Catholic University of Rio Grande do Sul, Porto Alegre, Brazil; Faculty of Exact and Natural Sciences, University of Antioquia, Medellín, Colombia; Department of Biological Sciences and Biodiversity, University of Los Lagos, Osorno, Chile; Institute of Ecology and Biodiversity, Concepción, Chile; Cape Horn International Center, Puerto Williams, Chile; Andean Cat Alliance, Villa Carlos Paz, Argentina; School of Health and Life Sciences, Pontifical Catholic University of Rio Grande do Sul, Porto Alegre, Brazil; Department of Biological Sciences, University of Caldas, Manizales, Colombia; Centro de Museos, Museo de Historia Natural, University of Caldas, Manizales, Colombia; Department of Biology, University of El Rosario, Bogotá, Colombia; Veterinary Integrative Biosciences, Texas A&M University, College Station, TX, USA; Interdisciplinary Program in Genetics & Genomics, Texas A&M University, College Station, TX, USA; Department of Biology, University of Antwerp, Antwerp, Belgium; Naturalis Biodiversity Center, Leiden, Netherlands; School of Health and Life Sciences, Pontifical Catholic University of Rio Grande do Sul, Porto Alegre, Brazil; Instituto Pró-Carnívoros, Atibaia, Brazil

**Keywords:** felidae, demographic history, genetic diversity, heterozygosity, introgression, neotropics, phylogenomics, phylogenetic discordance, radiation, runs of homozygosity

## Abstract

Even in the genomics era, the phylogeny of Neotropical small felids comprised in the genus *Leopardus* remains contentious. We used whole-genome resequencing data to construct a time-calibrated consensus phylogeny of this group, quantify phylogenomic discordance, test for interspecies introgression, and assess patterns of genetic diversity and demographic history. We infer that the *Leopardus* radiation started in the Early Pliocene as an initial speciation burst, followed by another in its subgenus *Oncifelis* during the Early Pleistocene. Our findings challenge the long-held notion that ocelot (*Leopardus pardalis*) and margay (*L. wiedii*) are sister species and instead indicate that margay is most closely related to the enigmatic Andean cat (*L. jacobita*), whose whole-genome data are reported here for the first time. In addition, we found that the newly sampled Andean tiger cat (*L. tigrinus pardinoides*) population from Colombia associates closely with Central American tiger cats (*L. tigrinus oncilla*). Genealogical discordance was largely attributable to incomplete lineage sorting, yet was augmented by strong gene flow between ocelot and the ancestral branch of *Oncifelis*, as well as between Geoffroy's cat (*L. geoffroyi*) and southern tiger cat (*L. guttulus*). Contrasting demographic trajectories have led to disparate levels of current genomic diversity, with a nearly tenfold difference in heterozygosity between Andean cat and ocelot, spanning the entire range of variability found in extant felids. Our analyses improved our understanding of the speciation history and diversity patterns in this felid radiation, and highlight the benefits to phylogenomic inference of embracing the many heterogeneous signals scattered across the genome.

## Introduction

Dissecting the evolutionary history of radiating lineages can be informative about the underlying evolutionary processes that generate diversity (Naciri and Linder 2020), yet often remains a difficult task even with whole-genome sequence (WGS) data available. Even when applying a phylogenetic framework under the assumption of the multispecies coalescent (MSC) model (Degnan and Rosenberg 2009), confident reconstruction of the species tree in a radiation is often hampered by the presence of large amounts of genealogical discordance (Bravo et al. 2019; Murphy et al. 2021). In addition to methodological limitations, such discordant phylogenetic signals are the consequence of two biological phenomena. First, incomplete lineage sorting (ILS) of ancestral genetic variation is common under a scenario of multiple speciation events that proceed in quick succession, i.e. the initial stages of a radiation. Therefore, ILS is typically invoked as the default cause of phylogenomic incongruence (Hibbins and Hahn 2022). Second, hybridization can lead to the introgression of genetic material from one lineage into another. In the context of a radiation, hybridization events may be prevalent because of incomplete reproductive isolation between emerging lineages (Harrison and Larson 2014). In addition, the influx of novel genetic material can potentially boost the evolvability of the recipient lineage (Gillespie et al. 2020). Since introgression leads to truly reticulate phylogenetic relationships, it contributes to phylogenomic incongruence. However, in many cases, its effect on diversity patterns may be difficult to distinguish from both speciation and ILS (Mallet 2005). To fully appreciate the evolutionary history of a radiation, it becomes necessary to quantify and carefully characterize any observed phylogenomic discordance (Bravo et al. 2019; Murphy et al. 2021).

The faunal exchange between North and South America induced by the formation of the Panamanian Isthmus provided many species with an opportunity to expand and diversify on a continental scale (Simpson 1980). One of the migrating clades that arrived and thrived in South America was the felid genus *Leopardus*, all of whose extant species are endemic to the Neotropical realm. The genus diverged over the course of the last 4.6 million years (credibility interval between 3.3 and 5.2 million years) (Trindade et al. 2021), with multiple speciation events taking place after the height of the Great American Biotic Interchange (GABI) 2.6 million years ago (mya) (Cione et al. 2015). *Leopardus* species now occupy a broad range of habitats, spanning from chaparral areas in Texas to shrublands in Patagonia, from high altitude Andean salt flats to lowland riverine rain forest (Wilson and Mittermeier 2009), and their geographic ranges often overlap (Kitchener et al. 2017). The exact taxonomy of *Leopardus* is subject to ongoing discussion. The genus is composed of five unanimously accepted species and 2 additional species complexes, totaling 8 (Kitchener et al. 2017) or up to 13 distinct species (Nascimento and Feijó 2017; Nascimento et al. 2020). Other basic aspects of the evolutionary history of this species-rich and ecologically diverse clade of felids also remain unclear, including its history of speciation, occurrence, and timing of interspecies hybridization, and other demographic events that shaped present-day population sizes and genetic diversity.

Recent genome-wide phylogenies based on single-nucleotide polymorphism (SNP) data (Li et al. 2016; Trindade et al. 2021) revealed discrepancies with previous studies that were based on markers with a limited genomic scope, including mitochondrial DNA (mtDNA), microsatellites, indels, short interspersed nuclear elements, supermatrices of selected genes, and the nonrecombining section of the Y-chromosome (Johnson et al. 1999; Pecon-Slattery et al. 2004; Johnson et al. 2006). Furthermore, these previous SNP phylogenies either lacked the southern tiger cat (*L. guttulus*) (Li et al. 2016) or showed that the Andean cat (*L. jacobita*) had an unstable position likely affected by missing data (Trindade et al. 2021). As such, no genome-wide analysis is currently available that includes the eight *Leopardus* species recognized by the Cat Specialist Group of the International Union for the Conservation of Nature (IUCN CSG) (Kitchener et al. 2017). A subsequent genomic analysis revealed very high fractions of topological discordance along the genome of four *Leopardus* species, more so than in any other felid lineage (Li et al. 2019). The extent of this discordance explains the difficulties in reconstructing the speciation history in *Leopardus* and calls for a comprehensive phylogenomic analysis.

Given the rapid diversification and, as a consequence, the short internal branches in the phylogeny of the ocelot lineage, it is likely that large portions of the genome are subject to ILS (Hibbins and Hahn 2022). In addition, introgressive hybridization has been amply demonstrated among *Leopardus* species: (i) a contemporary hybrid zone exists at the range edges of the southern tiger cat (*L. guttulus*) and Geoffroy's cat (*L. geoffroyi*); (ii) mtDNA of pampas cat (*L. colocola* sensu Kitchener et al. 2017) has been captured by a northeastern Brazilian population of the northern tiger cat (*L. tigrinus*), here referred to as a subspecies, the eastern tiger cat (*L. tigrinus emiliae*—but see Nascimento and Feijó (2017), who have proposed its elevation to species, *L. emiliae*); and (iii) the ocelot (*L. pardalis*) has potentially hybridized with a population ancestral to the clade containing the northern tiger cat (*L. tigrinus*) and Geoffroy's cat (*L. geoffroyi*) (Johnson et al. 1999; Trigo et al. 2008; Trigo et al. 2013; Ramirez et al. 2022). Like ILS, introgression events have likely added to the complexity of the evolution of this genus. Therefore, accurate inference of the species involved as well as the extent of these introgression events becomes crucial to fully understand the genus’ history.

The genetic diversity and demographic history of each *Leopardus* species are known to varying extents (e.g. Eizirik et al. 1998; Napolitano et al. 2008; Cossíos et al. 2012; Napolitano et al. 2014; Ruiz-García et al. 2018; Santos et al. 2018). Previous studies have used a variety of different data types to obtain diversity estimates, making it hard to directly compare published estimates. Even where diversity estimates are compared on identical methodological grounds, it is clear that species vary remarkably from one to the other. For example, an initial genomic survey of some *Leopardus* species indicated that the ocelot (*L. pardalis*) has the highest autosomal heterozygosity of any extant, wild felid reported so far, more than five times as diverse as the congeneric northern tiger cat (*L. tigrinus*) (Ramirez et al. 2022). These initial results highlight the need to perform detailed demographic analyses covering all species to understand what processes gave rise to this large variation in present-day genetic diversity.

In this study, we query whole-genome sequencing data (including novel WGS data for 9 samples reported here) of all *Leopardus* species recognized by the latest IUCN taxonomy source (Kitchener et al. 2017) to address how speciation, hybridization, and demographic events shaped taxonomic and genetic diversity. We determine the history and timing of speciation and characterize topological discordance within the genus. To detect past and ongoing hybridization events, we perform genome-wide introgression tests. Lastly, we infer historic population sizes for each species and estimate genomic divergence, levels of heterozygosity, and runs of homozygosity (runs of homozygosity [ROHs]).

## Results

### Quality Control, Mapping, and Base Calling

Our WGS data set with 15 *Leopardus* samples (supplementary table S1, Supplementary Material online) yielded good quality reference alignments, consensus genomes, and variant call sets for all subsequent genomic analyses. Of the 300+ million raw reads per sample, 85% to 95% had a phred quality score of 30 or higher (supplementary table S2, Supplementary Material online). After filtering the reads and mapping each sample to the 2.4 Gbp Canada lynx (*Lynx canadensis*) reference genome, the resulting effective sequencing depth varied between 17 and 26×, excluding the puma (*Puma concolor*) outgroup sample at 34×. We called bases from the Binary Alignment Map (BAM) files for each sample to retrieve pseudohaploid consensus genomes, which we masked for the 44% of repetitive content found in the reference genome. The remaining bases in the consensus genomes covered 82% to 95% of the nonrepetitive part of the reference (supplementary table S3, Supplementary Material online). The consensus genomes were aligned and split into nonoverlapping genomic fragments (GFs) with a length of 100 kb, yielding 24,078 GFs. A set of 16,338 GFs (68%) remained after removing all alignments in which at least one sample had more than 60% missing data. These GFs contained on average 55k unmasked, nonmissing base pairs (bp) (range 42-84k) of which, on average, 4,306 sites were informative for phylogenetic reconstruction (range 2,156-15,384). In addition to GFs from consensus genomes, we also created a variant call set from the BAM files, calling 15-17 million nonreference SNPs per sample (supplementary table S3, Supplementary Material online), or close to one SNP for every 150 bp in the genome on average. After masking regions with repetitive content in the reference genome, 9 to 10 million SNPs remained per sample. For mapping and base calling details with Geoffroy's cat reference, see supplementary text and supplementary table S4, Supplementary Material online.

### Mitogenomic Phylogeny

**Fig. 1.**
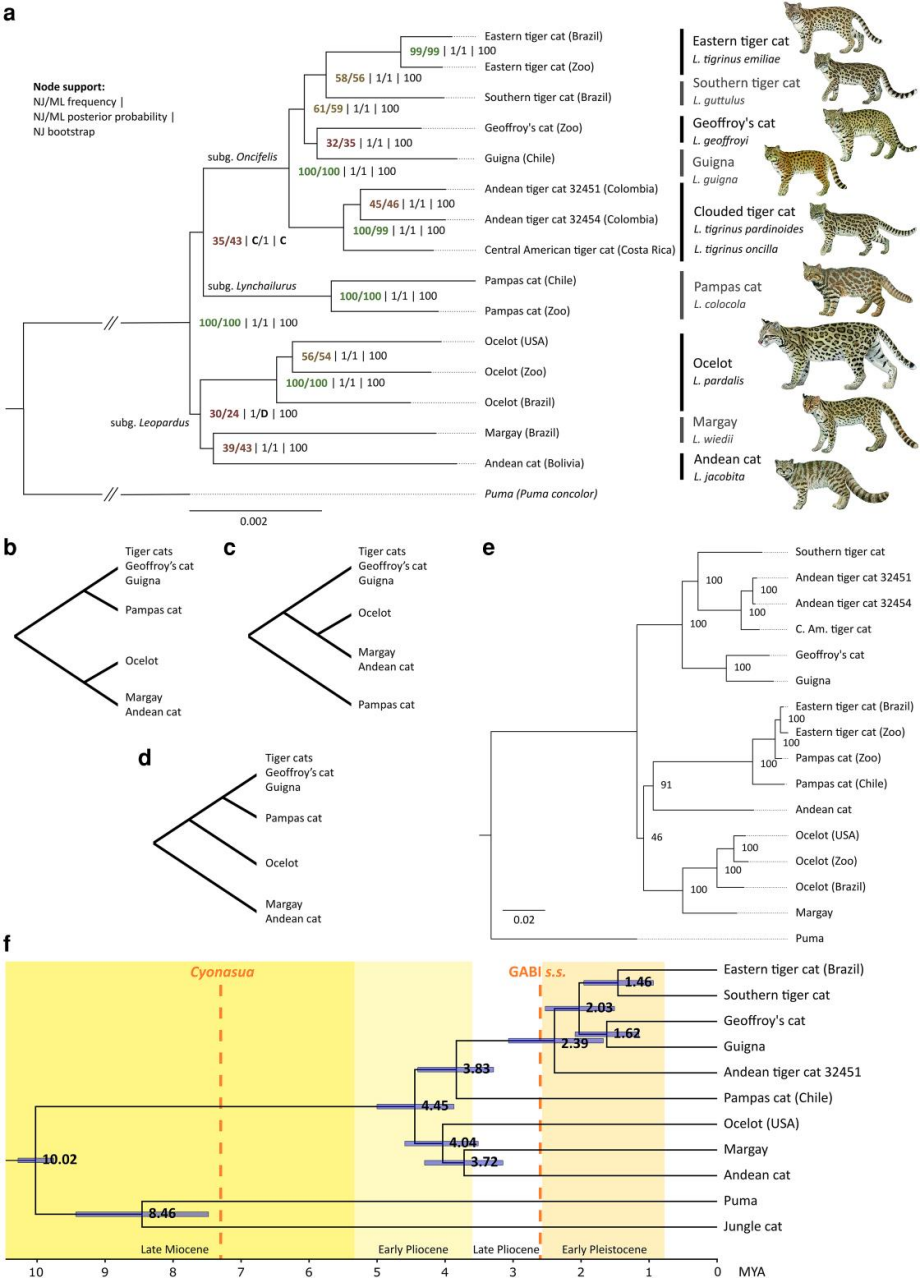
a) Greedy consensus phylogeny of 16,338 local NJ trees, annotated with node support across multiple methods for local tree inference and summary. Nodes are annotated with the node frequency support in the NJ and ML greedy consensus trees, local posterior probability (PP) support in the NJ and ML ASTRAL species trees, and percentage bootstrap support in the NJ tree derived from the whole-genome distance matrix. Nodes that are discordant between summary methods are indicated with a letter referring to their alternative topology. An illustration of each species [by T. Llobet (© Cornell Lab of Ornithology)] is shown next to its name; for geographic ranges of these felids, see Kitchener et al. (2017). b) Topology depicted in (a) and obtained with most summary methods and different analytical approaches (see also supplementary figs. S1 to S3, Supplementary Material online). c and d) Alternative topologies. e) Mitogenomic phylogeny with percent bootstrap support. f) MCMCTree consensus estimates for species-level divergence times from 16,186 dated, local trees. Nodes are annotated with their average divergence time estimate, and bars indicate the 95% HPD interval. Boundaries of geochronological epochs follow the International Chronostratigraphic Chart (Cohen et al. 2013). *Cyonasua*, an extinct genus of procyonids, represents the earliest southward mammalian migration of the GABI, 7.3 mya (Woodburne 2010). GABI *s.s.* (sensu stricto) indicates the height of the faunal exchange between the American continents, 2.6 mya (Cione et al. 2015).

The mitogenomic phylogeny was in accordance with previously published mitochondrial trees (Li et al. 2016), with additional placement of the southern tiger cat as well as the Andean population of the northern tiger cat (Fig. 1e). The assembled mitogenomes did not contain any missing data and varied in length between 17,197 and 17,944 bp, compared to 17,044 bp in the Canada lynx reference. The alignment of all 16 mitogenomes contained 9% gaps and 1,810 informative sites for the maximum likelihood (ML) analysis. The best-scoring ML tree was fully congruent with the mitogenomic phylogeny reported by Li et al. (2016), but did contain a basal polytomy (bootstrap support <70%). The mitochondrial DNA supported (i) a sister relationship of ocelot and margay, (ii) a nested position for the eastern tiger cat within pampas cat, (iii) Andean cat in a basal association with pampas cat, and (iv) Geoffroy's cat and guigna as sister species, next to a group comprising the southern tiger cat and the Andean/Central American populations of the northern tiger cat.

### Genomic Phylogeny

We retrieved a consistent topology across most combinations of phylogenetic methods and input data. Neighbor-joining (NJ) and ML phylogenetic reconstruction of genome-wide, masked, 100 kb-sized GFs aligned to the Canada lynx reference resulted in two sets of 16.338 local trees. The greedy consensus trees of the NJ and ML sets converged on the same topology, while the ASTRAL species trees and the genome-wide NJ tree deviated in either the position of the ocelot or the pampas cat (Fig. 1a to d). The greedy consensus topology received additional support by all but one phylogenetic analysis (ML ASTRAL) conducted with unmasked versions of the genomes (supplementary fig. S1, Supplementary Material online) and by all but one analysis when we mapped our data to an ingroup Geoffroy's cat reference genome instead of the Canada lynx reference (supplementary fig. S2, Supplementary Material online).

ASTRAL reconstructions received full concordance factor (CF) support, whereas node frequency support in the greedy consensus summaries was generally low (<60%) as would be expected in the presence of ILS (Smith et al. 2015) or ancestral gene flow. Node frequency support was slightly higher in the ML consensus compared to the NJ consensus, indicative of more noise in the NJ tree set. Despite generally low node frequency support, species-level clades with multiple individuals (ocelot, pampas cat, eastern tiger cat, and Andean/Central American tiger cats) achieved near-complete genome-wide support (>98%). The northern tiger cats (*L. tigrinus*) were an exception in this regard. The paraphyly of tiger cats, due to the position of the Central American tiger cat (*L. tigrinus oncilla*), basal to a clade containing Geoffroy's cat (*L. geoffroyi*), guigna (*L. guigna*) and other tiger cats (*L. tigrinus* and *L. guttulus*), had been demonstrated in previous studies (Li et al. 2016; Trindade et al. 2021) and was unambiguously supported in all our whole-genome phylogenies. The newly sequenced Andean tiger cats (*L. tigrinus pardinoides*) formed a well-supported monophyletic group with the Central American tiger cat, while the remaining northern tiger cats made up a distinct, equally well-supported clade, i.e. the eastern tiger cat (*L. tigrinus emiliae*). In contrast to other nodes above the species-level, full frequency support was attained for the monophyletic grouping that contains all tiger cats, Geoffroy's cat, and guigna. We propose to refer to this clade as the subgenus *Oncifelis* (see Discussion). Surprisingly, Andean cat was consistently retrieved as sister species with margay, challenging the long-held notion that margay and ocelot are sister species.

### Divergence Time Estimation

MCMCTree divergence time estimates indicated that crown group *Leopardus* started diversifying in the Early Pliocene (5.33 to 3.60 mya), with a basal split estimated at 4.45 mya (Fig. 1f and supplementary table S5, Supplementary Material online). Multiple speciation events followed in the span of <1 my, giving rise to the progenitors of ocelot (4.04 mya), margay/Andean cat (3.72 mya) and pampas cat/*Oncifelis* (3.83 mya). Subgenus *Oncifelis* in turn diversified over the course of the Early Pleistocene (2.58-0.77 mya), after the height of the GABI (2.6 mya). The lineage leading to the Andean and Central American tiger cat populations was the first to establish (2.39 mya), with the remainder of the clade subsequently developing as two pairs of sister species that include the Geoffroy's cat and guigna (1.62 mya) and the southern and eastern tiger cat at (1.46 mya). We computed the relative cross-coalescent rate (rCCR), along with a related Isolation-Migration (IM) approach, for the more recently separated populations of Central American and Andean tiger cats, and obtained point estimates for their split time at 157 kya (rCCR) and 171 kya (IM) (supplementary fig. S4, Supplementary Material online). Multiple variations of the IM approach yielded a range of estimates between 176 and 81 kya (supplementary figs. S5, Supplementary Material online).

### Phylogenomic Discordance

**Fig. 2.**
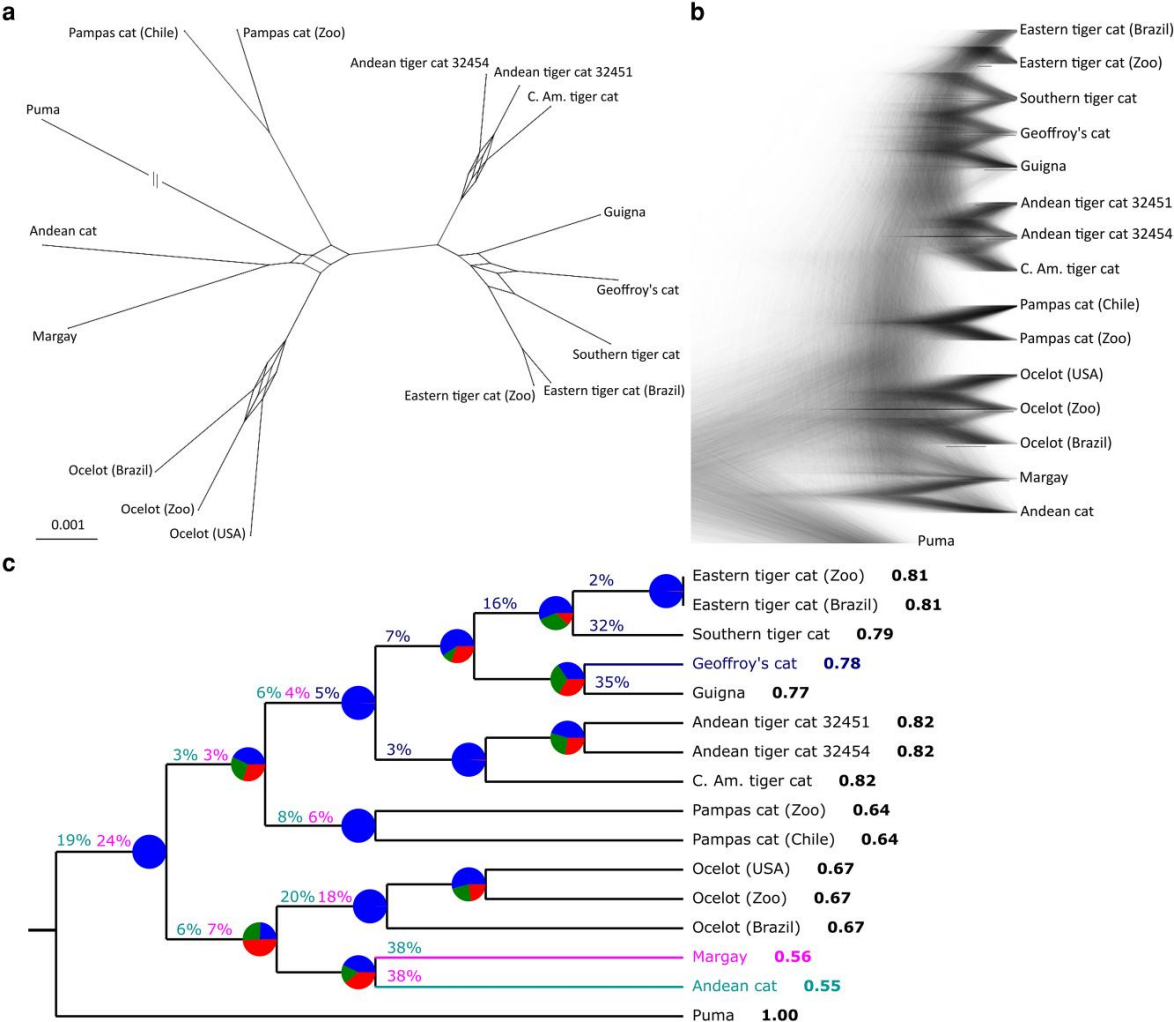
a) Implicit consensus network of a subset (20%, or 3,268 trees) of the ML tree set with a minimum threshold of 20% for inclusion of edges. b) DensiTree overlay of all 16,338 local ML trees. C) Consensus topology with node-specific conflict as pie charts, the LSI to the right of each sample, and color-coded branch attachment frequencies for Geoffroy's cat (blue), margay (pink) and Andean cat (teal). Pie charts show the proportion of local trees that contain the same clade as the consensus tree in blue, the proportion of trees that contain the most common alternative in green, and the total proportion of all remaining alternatives in red. Clades with only blue are monophyletic in all local trees, clades with a high fraction of green have a single dominant alternative configuration, and clades with a higher proportion of red than green are highly unstable throughout the tree set. The branch attachment frequencies were counted using a pruned sample set (see supplementary fig. S6, Supplementary Material online).

We found extensive phylogenetic discordance among local trees. For 15 samples and a fixed outgroup, we recorded 7,983 distinct topologies in the ML tree set, with the most frequent topology appearing only 80 times, or 0.005% of the 16,338 local trees. Discordance among local ML trees is visualized as an overlay of all trees (Fig. 2b) and as an unrooted Median Consensus Network (Fig. 2a). The implicit network included 37 splits and 67 edges and shows that the majority of phylogenomic discordance is situated at the base of the genus, at the base of *Oncifelis*, and between triplets of conspecific samples. Results with local NJ trees display slightly higher discordance (supplementary fig. S7, Supplementary Material online).

**Fig. 3.**
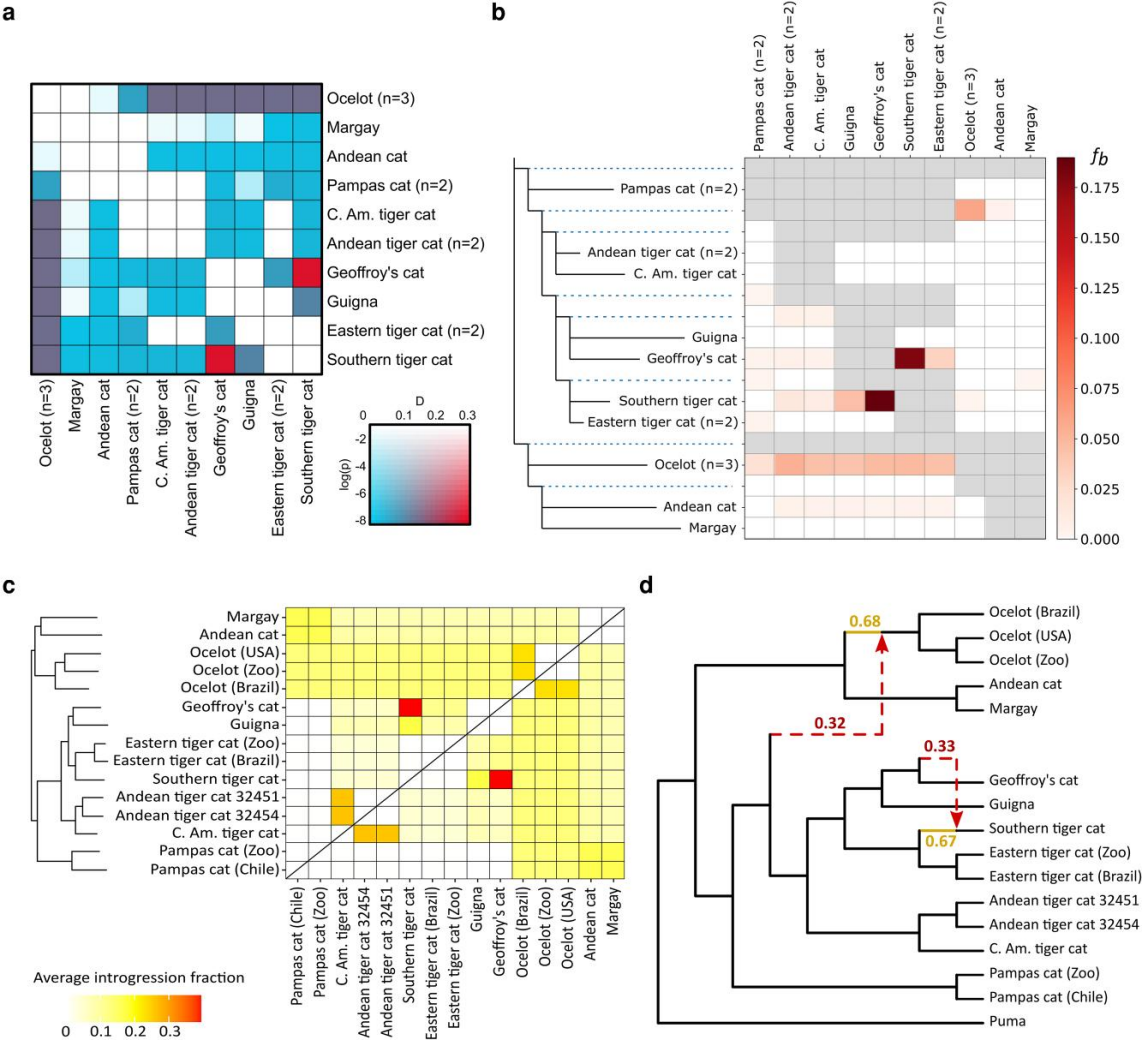
a) Genome-wide pairwise Patterson's D, calculated from ABBA/BABA site patterns in the SNP data set. Colors correspond to statistical support (light to dark) and magnitude (blue to red) of the D-statistic. b) Pairwise *f*-branch (*fb*) statistic as measure of the fraction of introgression between extant and ancestral populations or species, inferred from the SNP data set. c) Pairwise average fractions of introgression between samples inferred from branch length information in 16,338 local ML trees. d) Optimal phylogenetic network inferred under a maximum of 2 hybrid edges. d) The best-scoring phylogenetic network (log-pseudolikelihood = −189.36) after 50 iterations and restricted to 2 hybrid edges, inferred from the topologies of 16,338 local ML trees. See supplementary fig. S12, Supplementary Material online for selection of the number of hybrid edges.

Node-specific conflict, projected on the consensus tree, was high for several nodes, and several terminal taxa were highly unstable in their position (Fig. 2c). In accordance with the greedy consensus tree, species-level nodes and the node grouping of all *Oncifelis* samples proved very stable. Other nodes allowed for alternative intraspecific placements (among ocelot samples or Andean/Central American tiger cat samples) or allowed for other alternatives (e.g. margay and Andean cat). The margay and Andean cat were the most unstable taxa, as shown by their low Leaf Stability Index (LSI). To explore the samples’ alternative positions observed in the tree set, we counted their branch attachment frequency (BAF) and highlighted the frequency of alternative attachments for selected taxa (Fig. 3c, see supplementary fig. S6, Supplementary Material online for all samples). The Andean cat and margay were only placed in a sister-species position in 38% of the local trees, and both were frequently associated with ocelot or had a basal position in the genus. Geoffroy's cat occupied its traditional sister position with guigna in 35% of trees, attaching in almost equal measure (32%) to the southern tiger cat, and to a lesser degree attaching to the ancestral branch of southern tiger cat and eastern tiger cat.

### Introgression

We recovered high gene flow between Geoffroy's cat and southern tiger cat as well as historical introgression between ocelot and the root of the *Oncifelis* clade in all introgression tests. In our first test, we computed genome-wide D-statistics with Dsuite to infer introgression from imbalances in ABBA/BABA site patterns. Patterson's D (Fig. 3a) and the *f_4_*-ratio (supplementary fig. S8, Supplementary Material online) were elevated for many species comparisons. To reduce false-positive signals in comparisons with a focal recipient species caused by genetic similarity of species related to the donor species, we also computed the *f*-branch statistic (Fig. 3b). The estimated admixture fraction was largest between Geoffroy's cat and southern tiger cat (18%) and another strong signal of admixture was found between ocelot and the ancestral branch of *Oncifelis* (6%). D-statistics remained consistent when samples were mapped to the ingroup Geoffroy's cat reference (supplementary figs. S9 to S11, Supplementary Material online).

Our second approach, the QuIBL test for introgression, was based on the distribution of branch lengths in a random 20% subsample of the tree set. It confirmed a high fraction of gene flow between Geoffroy's cat and the southern tiger cat (40%) (Fig. 3c and supplementary table S6, Supplementary Material online). Estimates of intraspecific gene flow between the three ocelot samples were high (23%) and comparable among the Andean tiger cat samples and the Central American tiger cat (26%). Shared ancestry between ocelot and the *Oncifelis* species, but also with the pampas cat, was estimated at 13%.

Lastly, based on topological imbalance in the set of 16,338 local ML trees, we inferred explicit phylogenetic networks under a maximum pseudolikelihood model with PhyloNetworks. The slope heuristic identified h = 2 as the optimal number of reticulations (supplementary fig. S12, Supplementary Material online). The best network with two reticulations (log-pseudolikelihood −189.36) inferred a large fraction of introgression from Geoffroy's cat into the southern tiger cat (33%) and from a population ancestral to *Oncifelis* into the ocelot (32%) (Fig. 3d). This finding remained consistent when samples were mapped to the ingroup Geoffroy's cat reference (supplementary fig. S13, Supplementary Material online).

### Genomic Diversity

**Fig. 4.**
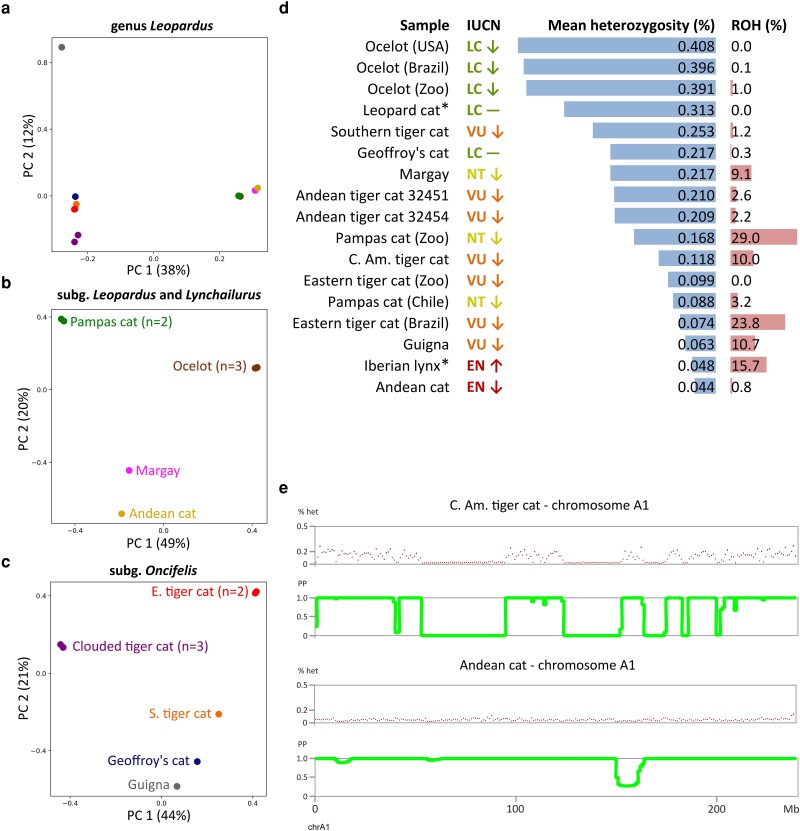
a) PCA plot for all samples in the genus *Leopardus*, computed from the SNP data set with the Geoffroy's cat reference (507,590 SNPs after pruning). Samples are color-coded as in b and c. b) PCA plot including samples of subgenera *Leopardus* (ocelot, margay, Andean cat) and *Lynchailurus* (pampas cat). c) PCA plot of samples in subgenus *Oncifelis* (tiger cats, guigna, Geoffroy's cat). d) Average autosomal heterozygosity and autosomal ROH content per sample estimated with ROHan, annotated with IUCN extinction risk status and population trend (decreasing, stable, or increasing). Samples indicated with an asterisk are from other felid genera and were included for context. All samples were mapped to the Geoffroy's cat reference, except the Iberian lynx which was mapped to its congeneric Canada lynx reference to improve the estimates. See supplementary tables S9 to S10, Supplementary Material online for heterozygosity estimates using multiple methods and different reference genomes. e) Local heterozygosity and Bayesian inference of ROHs along chromosome A1 in a sample with high ROH content “C. Am. tiger cat” and a sample with low ROH content “Andean cat”. The x-axis shows the chromosomal coordinates, the y-axis show either percentage heterozygosity (% het) or PP. Regions with a low PP are identified as ROHs.

We assessed the genetic structure in the sample set with principal component analysis (PCA) and estimated indices of genetic diversity. After pruning for linkage disequilibrium, we retained 507,590 SNPs as input for the PCA. The first principal component captured 38% of the variation and mainly separated the *Oncifelis* clade from the other taxa. As expected, samples of the same species were tightly clustered (Fig. 4a). Additional PCAs (Fig. 4b and c), each restricted to the samples of only one of these two main groups, reflected findings from the phylogenetic and introgression analyses: margay was found closest to Andean cat, Central American tiger cat clustered closely with Andean tiger cat, and Geoffroy's cat and southern tiger cat were found intermediate between guigna and eastern tiger cat. These results were also obtained when samples were mapped to the ingroup Geoffroy's cat reference (supplementary fig. S14, Supplementary Material online).

Genome-wide average nucleotide diversity (*π*) varied widely across species, ranging from 0.14% in eastern tiger cats (n = 2) to 0.45% in ocelots (n = 3) (supplementary table S7, Supplementary Material online). Divergence ($D_{xy}$) between samples of different species of *Leopardus* averaged 0.67% and dropped to 0.41% among species in the *Oncifelis* clade, lower than the 0.45% intraspecific diversity observed in ocelot (supplementary table S7, Supplementary Material online). Sequence divergence was especially low (0.36%) between southern tiger cat and both eastern tiger cat and Geoffroy's cat, and highest (0.82%) between Andean cat and the pampas cat sample from Chile. Divergence from the outgroup puma sample averaged 1.72%. Estimates between samples were consistently elevated when we did not mask repetitive elements (supplementary table S7, Supplementary Material online). Genomic divergence between samples was roughly one order of magnitude lower than divergence between their mitochondrial sequences (supplementary table S8, Supplementary Material online).

Within-sample diversity, measured as autosomal heterozygosity, was largely consistent between intraspecific samples (except pampas cat), but highly variable between species (Fig. 4d). Autosomal average heterozygosity estimated with ROHan was extremely low in Andean cat (<0.05%, comparable to Iberian lynx, *Lynx pardinus*), low to intermediate in guigna, eastern tiger cat, pampas cat and Central American tiger cat (0.05% to 0.2%), high in Andean tiger cat, margay, Geoffroy's cat and Southern tiger cat (0.2% to 0.3%), and very high in ocelots (>0.3%). The average heterozygosity in the most diverse ocelot was almost tenfold higher than in the congeneric Andean cat. The relative position of each sample, ranked by heterozygosity, remained largely consistent when using another reference genome and across different methods to assess heterozygosity (supplementary tables S9 to S10, Supplementary Material online).

The genomes of several samples contained long runs of homozygosity (ROHs). The proportion of the genome identified as ROHs was highest in a captive individual, “Pampas cat (Zoo)” (Fig. 4d). High ROH content was also detected in wild-caught eastern tiger cat (23%), guigna (11%), Central American tiger cat (10%), and margay (9%) (Fig. 4d to e). In all samples, ROHs varied in length and could occur anywhere in the genome (supplementary figs. S15 to S16, Supplementary Material online).

**Fig. 5.**
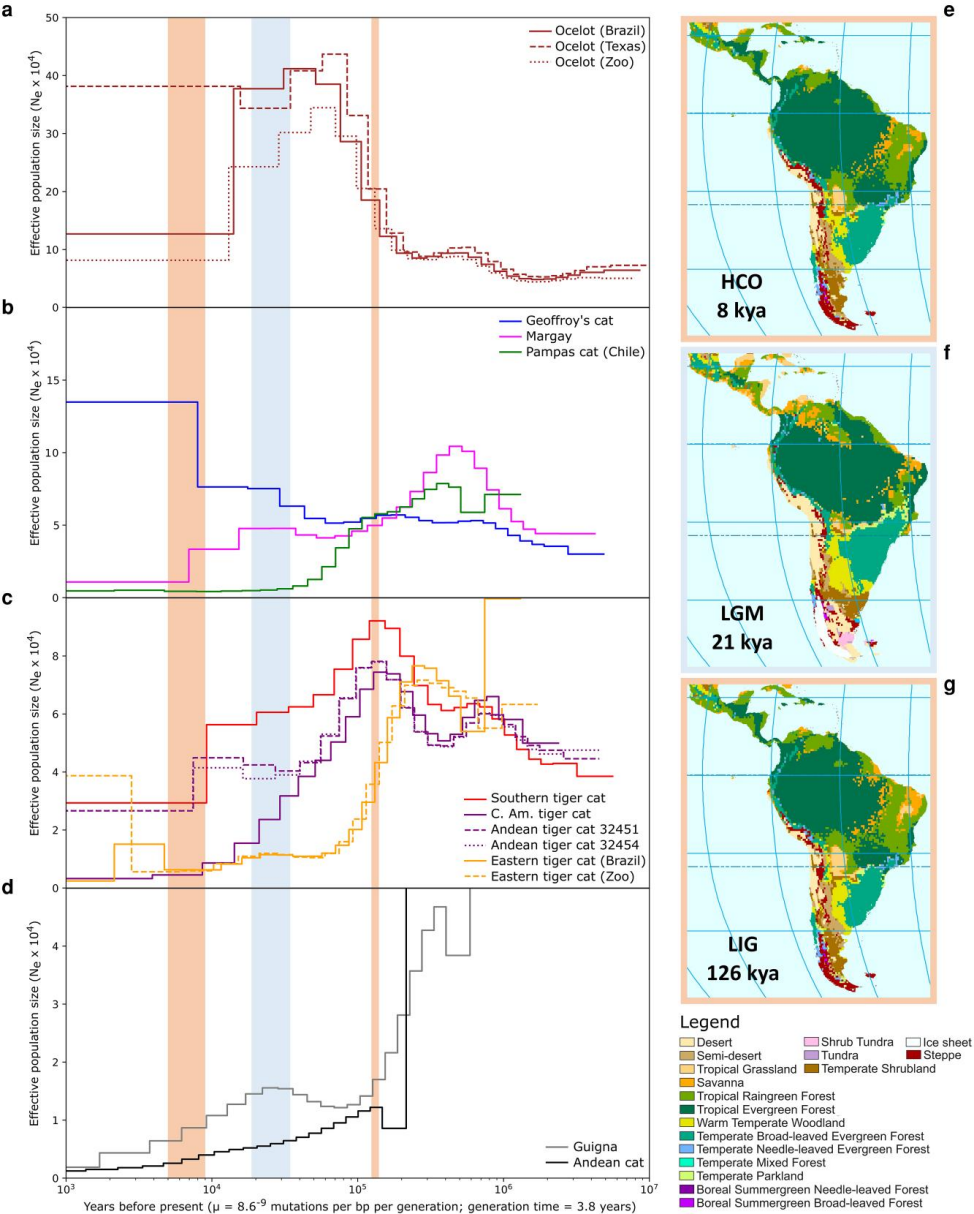
a to d) Demographic history of each *Leopardus* sample, mapped to the geoffroy's cat reference. The x-axis shows the coalescent time in years on a log scale, using a mutation rate μ = 8.6 × 10^−9^ and a generation time of 3.8 yr (Wang et al. 2022). The y-axes show the effective population size (*N_e_*). Note that maximum *N_e_* values vary across panels, between *N_e_* = 50,000 to *N_e_* = 500,000. Red and blue background colors denote climatic maxima and minima over the past 200 ky: the Holocene Climatic Optimum (HCO: 9-5 kya), the LGM (33-18 kya) and the LIG (130-115 kya). e to g) Reconstruction of paleovegetation in Central and South America during specific time points representing the climatic extremes in the HCO, LGM, and LIG. Reproduced with permission from Allen et al. (2020).

We inferred highly variable effective population sizes (*N_e_*) over time, with alternating periods of population growth and decline in most species except for the Andean cat and the Central Chilean population of pampas cat. Different *Leopardus* species have reached vastly different maxima over the past 1 my (Fig. 5). From a maximum *N_e_* before or during the Last Interglacial (LIG: 130-115 kya), species such as the guigna, Andean cat, margay and southern tiger cat as well as specific populations of northern tiger cat (Costa Rica, NE Brazil) and pampas cat (Chile) have declined to much lower levels in the Holocene (11.7 kya—present). While this decline has been continuous in Andean cat, Chilean pampas cat, Central American tiger cat, and southern tiger cat, others experienced an uptick in *N_e_* during either the Last Glacial Maximum (LGM: 33-18 kya) (margay, guigna, and eastern tiger cat) or toward the Holocene (Andean tiger cat, and eastern tiger cat). Geoffroy's cat went through multiple cycles of population expansion and retraction, with the latest expansion initiated toward the LGM and achieving its highest estimate in the Holocene. The largest ancestral *N_e_* was observed in ocelot, which saw a fourfold increase in population size between ∼200-70 kya, before declining to pre-LIG levels in the Holocene. For the most recent time interval, the ocelot sample from Texas did not share the steep decline in estimated *N_e_* observed in the other two ocelot samples. Other populations with two samples such as the Andean tiger cat and the eastern tiger cat showed highly consistent results between samples and bootstrap replicates indicated reliable estimation of *N_e_* in all but the oldest time interval (supplementary fig. S17, Supplementary Material online), strengthening our confidence in the accuracy of the method. The lowest *N_e_* was observed in Andean cat, which has been steadily declining in effective population size since at least the LIG.

## Discussion

### Definition of Subgenera and Phylogenetic Position of Andean Cat and Andean Tiger Cat

Our whole-genome consensus phylogeny (Fig. 1a) largely corroborates previous phylogenies obtained from SNP data (Li et al. 2016; Trindade et al. 2021), yet challenges the traditional view of margay and ocelot as sister species in favor of a closer relationship between margay and Andean cat. Placement of the latter has always been contentious. Preceding molecular studies proposed the species to be associated with pampas cat (Johnson et al. 2006) or at a basal position to an ocelot/margay sister pair (Johnson et al. 1998; Li et al. 2016). Instead, our nuclear phylogenies consistently recover Andean cat with margay, irrespective of the choice of reference genome, masking strategy, method for local tree inference and summary tree method (Fig. 1a, supplementary S1 to S3, Supplementary Material online). We, therefore, conclude that the tropical, highly arboreal margay, which shares its rosetted black-on-gold coat pattern and its mostly forested habitat with ocelot, is in fact more closely related to the Andean cat, a highland specialist with a distinct morphology that is somewhat reminiscent of a pampas cat. Strengthened by the new position of margay, we posit that the rosetted coat pattern seen in margay, ocelot, and tiger cats is the most parsimonious ancestral pelage phenotype in *Leopardus*. In that scenario, Andean cat and pampas cat would have independently attained their alternative coat patterns, while the rosettes evolved into spots in the common ancestor of guigna and Geoffroy's cat.

The Central American tiger cat confidently clusters with the previously unsampled Colombian tiger cat. Our two new tiger cat samples from Colombia represent the population of northern tiger cat found in the three cordilleras of the northern Andes (González-Maya et al. 2022), a population referred to as the Andean tiger cat (de Oliveira et al. 2022) or *L. tigrinus pardinoides* (Gray, 1867) *nom. dub.* (Nascimento and Feijó 2017). The tiger cat sample from Costa Rica represents the population of northern tiger cat in the Talamanca Mountain Range (Rodgers and Kapheim 2017), referred to as the Central American tiger cat or *L. tigrinus oncilla* (Thomas 1903) sensu Kitchener et al. (2017). The three samples are recovered in a monophyletic group across 99% to 100% of the local topologies (Fig. 1a), a clade that diverged 2.39 mya (95% HPD 3.07 to 1.67 mya) from the ancestral lineage of the other tiger cats, Geoffroy's cat and guigna (Fig. 1f). As argued previously (Trindade et al. 2021) and now reaffirmed by WGS data and additional samples, the Central American tiger cat and the Andean tiger cat, colloquially referred to as “clouded tiger cats” due to their irregular rosettes (de Oliveira et al. 2022) and elevated (>1,500 m) cloud forest habitat (Rodgers and Kapheim 2017; Cepeda-Duque 2022), should be assigned an appropriate taxonomic status as a distinct species. However, the exact taxonomic designation of the species currently contained in the paraphyletic taxon *L. tigrinus* depends on the phylogenetic position of tiger cats from the species’ type locality in the Guiana Shield region to which the name *L. tigrinus* (Schreber 1775) is attached. This type population remains molecularly unsampled, and it is therefore of the highest priority to obtain WGS data from tiger cats in the Guianas.

The genetic relationship between the populations of Andean and Central American tiger cat is more consistent with subspecies status than with species-level divergence. Levels of topological discordance (Fig. 2C) and the inferred fraction of intraspecific gene flow (Fig. 3c) are higher among the three samples of clouded tiger cat than among the three samples of ocelot. Conversely, nuclear and mitochondrial nucleotide diversity (*π*) are lower in our clouded tiger cat samples than in the conspecific ocelot samples (supplementary tables S7 to S8, Supplementary Material online). The populations of Andean and Central American tiger cat are believed to be currently isolated, with the Chocó-Darién moist forest ecoregion acting as a warm, lowland barrier that separates the colder, elevated habitat in the Andes and in the Talamanca Mountain Range (Bonilla-Sánchez, unpublished data). Based on the disparity in their demographic trajectories, their divergence most likely took place between 176-81 kya (supplementary fig. S4 to S5, Supplementary Material online). Being similar to the 112 kya split inferred for subspecies of Asian golden cat (*Catopuma temminckii*) (Patel et al. 2016), this divergence estimate fits within the observed range of subspecies-level splits in other felids, e.g. 67 kya in tigers (Liu et al. 2018) and an 500-600 kya upper age limit in leopards (Paijmans et al. 2021), and is an order of magnitude smaller than that between closely related cat species, e.g. 1.46 mya for eastern and southern tiger cat (Fig. 1f). To confidently assess the degree of distinction between Andean and Central American tiger cats, it will be necessary to perform population-level sampling and subsequent gene flow analyses along with ecological assessments of their distinctiveness.

For ease of reference and communication, we propose to distinguish three monophyletic subgenera in the genus *Leopardus* (Gray, 1842): (i) the nominotypical *Leopardus* subgenus *Leopardus* (Gray, 1842) with type species *Leopardus pardalis* (Linnaeus, 1758), which includes ocelot (*Leopardus pardalis*), margay (*Leopardus wiedii*) and Andean cat (*Leopardus jacobita*); (ii) *Leopardus* subgenus *Lynchailurus* (Severtzov, 1858) with type species *Leopardus colocola* (Molina, 1782), which includes the pampas cat species complex (*Leopardus colocola*); and (iii) *Leopardus* subgenus *Oncifelis* (Severtzov, 1858) with type species *Leopardus geoffroyi* (d’Orbigny & Gervais, 1844), which includes Geoffroy's cat (*Leopardus geoffroyi*), guigna (*Leopardus guigna*) and the tiger cat species complex (*Leopardus guttulus* and *Leopardus tigrinus* incl. *L. t. oncilla*, *L. t. pardinoides* and *L. t. emiliae*).

### Two Pulses of Speciation

Whole-genome divergence time estimates suggest a scenario of two distinct waves of speciation in the ocelot lineage. An initial radiation unfolded in the Early Pliocene, contemporaneous with a period of major uplifting along the Talamanca Mountain Range in Central America (Gräfe et al. 2002). Following a period of taxonomic stability in the Late Pliocene, a second radiation conceivably occurred after the height of the GABI and resulted in the current diversity of the *Oncifelis* clade on the South American continent (Fig. 1f), presumably having diversified across the continent along a north-south axis. The ocelot lineage may have dispersed to South America during or after the period of most active exchange between the two continents, 2.6 mya (GABI sensu stricto) (Cione et al. 2015), after the definite closing of the Isthmus of Panama (2.8 mya) (O’Dea et al. 2016) and coincidental with the arrival of other felids (Woodburne 2010). As such, our molecular dating fits a Neotropical radiation for genus *Leopardus* (Johnson et al. 2006; Li et al. 2016), but suggests that it may not have occurred entirely in South America. Our results refine the estimates obtained by Trindade et al. (2021) and suggest that this radiation may have started in Central America as the Isthmus of Panama was forming, followed by separate immigrations of newly diverged species in South America. At the same time, carnivoran colonization of South America by means of island hopping or intermittent land bridges has started as early as 7.3 mya with the successful immigration of procyonids (Woodburne 2010). We can therefore not exclude a single ancestral immigration event and a fully South American radiation for *Leopardus*, a scenario that has been supported recently for South American canids, a clade that was inferred to have radiated from a single ancestor that colonized South America 3.5 to 3.9 mya (Chavez et al. 2022). Regardless of absolute dating, a model of two distinct pulses of speciation events is supported by short internal branches (Fig. 1f) and high topological discordance (Fig. 2c) near the base of the genus and within the *Oncifelis* clade, with a long internal branch separating the well-supported *Oncifelis* from the other taxa.

### Prevalence of ILS and Interspecific Gene Flow

In phylogenomic data sets, topological variation across local trees is assumed to represent three distinct sorting histories: complete lineage sorting (speciation history), ILS and introgressive hybridization (Bravo et al. 2019; Hibbins and Hahn 2022). We accounted for ILS by inferring the species phylogeny in a two-step method that is consistent with the coalescent process, i.e. independent local tree estimation followed by distilling a summary tree from the set of local trees (Bravo et al. 2019). Common measures for node support such as bootstrap replicates or posterior probabilities reflect statistical precision rather than biological truth, and we, like others (e.g. Simon 2020; Singhal et al. 2021), find that the consensus phylogeny despite full node support may vary depending on specificities of the input data and inference methods (Figs. 1a to d and supplementary S1 to S3, Supplementary Material online). Therefore, we also included node frequency support, a measure that explicitly considers heterogeneity among local trees. The summary is then a greedy consensus, which is a composite tree constructed from the individual clades that attain the highest frequencies in the local tree set (i.e. a consensus that maximizes node frequency support). Node frequencies and similar measures such as spectral analysis or gene concordance factors offer an immediate impression of the topological discordance at each node (e.g. Hendy and Penny 1993; Smith et al. 2015; Vanderpool et al. 2020; Singhal et al. 2021), with ILS being the default explanation (Bravo et al. 2019; Hibbins and Hahn 2022). For example, alternative phylogenetic placement of margay and Andean cat seems to be driven by extensive ILS (>50%), a conclusion further supported by their balanced branch attachment frequencies (Fig. 2c) and the lack of a clear introgression signal between margay and ocelot (Fig. 3).

Interspecific gene flow has also contributed substantially to the phylogenomic discordance observed in *Leopardus* and may in part explain the difficulties in reconstructing the speciation history of the genus. The large fraction of material (18% to 40%) that was exchanged between the genomes of the southern tiger cat and Geoffroy's cat (Fig. 3b to d and supplementary table S6, Supplementary Material online) explains the high degree of phylogenomic discordance evident from the low frequency support values (32% to 37%) at the node grouping Geoffroy's cat with its sister species, the guigna (Fig. 1a, supplementary S1 to S3, Supplementary Material online). The disproportionally high BAF score (32%) for southern tiger cat and Geoffroy's cat at their hybrid position supports the inference of extensive gene flow and almost matches the fraction of the genome that conforms to the species tree (35%) (Fig. 2c). The southern tiger cat and Geoffroy's cat admix throughout a well-studied, contemporary hybrid zone found along the ecotone between Atlantic Forest and Pampas biomes in southernmost Brazil (Trigo et al. 2008, 2013, 2014; Sartor et al. 2021). The prevalence of naturally occurring *L. geoffroyi x L. guttulus* hybrid individuals is high, at the order of 14% to 18% in the region of range overlap (Trigo et al. 2008; Sartor et al. 2021), compared to the 7% to 8% hybrids between European wildcat (*Felis silvestris*) and domestic cat (*Felis catus*) (Pierpaoli et al. 2003; Tiesmeyer et al. 2020) and <1% hybrids between Canada lynx (*Lynx canadensis*) and bobcat (*Lynx rufus*) (Koen et al. 2014). In the specific context of the current, active hybrid zone, the samples of southern tiger cat and Geoffroy's cat used in this study have not been previously identified as hybrids (Sartor et al. 2021; Trindade et al. 2021). Therefore, either these species have had earlier hybridization events leading to introgression of genomic material that became fixed in the recipient population, or the active hybrid zone has a broader geographic scope than previously estimated with microsatellite data (Sartor et al. 2021) or with genome-wide SNPs from a small sample of individuals (Trindade et al. 2021).

An older hybridization event took place between the ocelot and the lineage ancestral to the *Oncifelis* clade, which currently contains the various tiger cat species, the guigna, and the Geoffroy's cat. Lasting signals of this event are found in 13% (branch length analysis, Fig. 3c) up to 32% of the genome (phylogenetic network analysis, Fig. 3d). These results are in agreement with previous indications from D-statistics with genomic data (Ramirez et al. 2022).

Our phylogeny from de novo assembled mitogenomes (Fig. 2e) reconfirms that the eastern tiger cat has assimilated pampas cat mtDNA (Trigo et al. 2013; Santos et al. 2018). As in the study by Trindade et al. (2021), we did not detect any trace of hybridization in the nuclear genome of these species. The mitochondrial topology is barely even present in the set of local genomic trees (0.05% BAF) (supplementary fig. S6, Supplementary Material online). ILS of the mitogenome cannot explain its nested position within the pampas cat, given that almost all of the nuclear genome managed to become sorted along the long internal branch that separates *Oncifelis* from the pampas cat (Fig. 2c). With that, mitochondrial capture without the retention of nuclear material remains the most likely scenario.

### Genetic Diversity and Demographic History

The various closely related species of *Leopardus* exhibit a remarkable disparity in estimates of divergence, heterozygosity, and historic trajectories of effective population size. For example, genome-wide levels of genetic divergence ($D_{xy}$) between species of the Early Pleistocene diversification of *Oncifelis* are lower than the intraspecific diversity (*π* and heterozygosity) observed in the ocelot, a species that originated in the Pliocene (Fig. 4d and supplementary table S7, Supplementary Material online) and possibly has the highest levels of diversity observed in any species of wild felid (Ramirez et al. 2022). The low levels of divergence in subgenus *Oncifelis* reflect the more recent origin and lower effective population sizes of these species compared to ocelot but additionally may have been diminished by postspeciation gene flow between Southern tiger cat and Geoffroy's cat. In terms of demographic history, the various species of *Leopardus* seemingly do not share a common pattern (Fig. 5). The demographic results echo each species’ particular distribution pattern and density across the Neotropics, although we cannot exclude the confounding effect of potential population structure and acknowledge that changes in *N_e_* may in fact represent changes in migration rates with unsampled populations rather than changes in census population size (Orozco-terWengel 2016).

The ocelot occurs at high densities and has the largest distribution in the genus (e.g. de Oliveira et al. 2022; Lombardi et al. 2022). It reaches the highest historic *N_e_* and harbors the most genetic diversity (Fig. 4d) of all *Leopardus* species. The segregating demographic trajectories of our three samples that start around the LIG could reflect the onset of population substructure across the species’ enormous range. Surprisingly, the most recent *N_e_* estimate for the individual from Texas is many times higher than that of the Brazilian ocelot. This finding runs contrary to expectations of low *N_e_* and census sizes (Janečka et al. 2008). Because of the coarse resolution, the observed difference in *N_e_* between Brazilian and US ocelots could reflect differential events at any time during the Holocene, such as the dramatic, human-mediated decrease in Brazilian Atlantic Forest habitat to 11% to 16% of its original cover (Ribeiro et al. 2009). Nonetheless, the US population has also decreased strongly in historic times, once reaching Arkansas and Louisiana (Haines et al. 2005). An unknown migration event of highly divergent alleles into the US population might also explain the high genetic diversity in our US sample.

At the other end of the spectrum stands the Andean cat. Restricted to a high-altitude habitat of rocky outcrops in the Andes and limited by low prey availability, it has been reported at low densities and with extremely low diversity in mitochondrial genes and nuclear microsatellites across its range (Napolitano et al. 2008; Cossíos et al. 2012). Low genetic diversity in the Andean cat is confirmed by low autosomal heterozygosity in our Bolivian sample (Fig. 4d) and seems to be a consequence of a slow but steady decline in population size since at least the LIG. Absence of ROHs in our sample indicates absence of inbreeding in the population from La Paz, Bolivia, and further supports a long-term small population size as the cause of low diversity, rather than recent, abrupt declines. Increased genomic sampling may determine whether this pattern is representative of all populations.

The demographic trajectories of most *Leopardus* samples span multiple periods of both population expansions and contractions over the past 1 myr (Fig. 5a to d). This cyclic pattern may be linked to Pleistocene glaciations, which have affected Neotropical temperature, humidity, and vegetation (Fig. 5e to g) in a complex patchwork of regional responses (Allen et al. 2020; Beyer et al. 2020). The most convincing correlation between population size and climatic shifts dictated by glacial cycles is found in the clouded tiger cat (*L. t. pardinoides* and *L. t. oncilla*). Since the maximum forest cover in the LIG (126 kya), the population size of this cloud forest specialist declined as both the northern Andes and Central America conceded sizeable regions of forest habitat to drier grassland (Woodburne 2010; Allen et al. 2020), which reached its maximum extent in the LGM (21 kya). Specifically in the Colombian Andes, the montane forest belt contracted during the LGM to make way for the expanding páramo alpine tundra (Hooghiemstra and Van der Hammen 2004). The Colombian population (*L. t. pardinoides*) again saw an uptick as forest habitat regained its footing toward the Holocene Climatic Maximum (8 kya) while the Central American population (*L. t. oncilla*) continued to decline until present, possibly because of its isolation in a restricted area of suitable habitat in the Talamanca Mountain Range. Lower heterozygosity and a higher fraction of ROHs in the genome of our Central American sample compared to the Colombian samples (Fig. 4d) further point to a higher degree of inbreeding and a lower population size.

At the latitudes of the southern Andes, populations on the western side of this geographical barrier are marked by lower historic population sizes, lower heterozygosities, and higher fractions of ROH than their eastern side counterparts. The Central Chilean pampas cat (*L. colocola colocola*) and the guigna (*L. guigna*) in Chile are confined to a much smaller range than the Geoffroy's cat (*L. geoffroyi*) east of the Andes (Napolitano et al. 2014; Nascimento et al. 2020) and arrived at similarly low present-day population sizes through different demographic histories. We recovered a historic population expansion for the northern subspecies of guigna (*L. g. tigrillo*) in the interval between the LIG (126 ya) and the LGM (21 kya) (Fig. 5d). This finding is consistent with results from a mismatch distribution analysis with mtDNA (Napolitano et al. 2014) and could relate to a northward expansion of the subspecies into central Chile, a common migration route for species that crossed the southern Andes from Argentine Patagonia (Moreno et al. 1994). Since the LGM, the population has been declining continuously. The Central Chilean pampas cat population instead experienced a strong bottleneck leading up to the LGM, from which it seemingly never recovered (Fig. 5b). Glacial ice sheet coverage in the southern Andes may have had a profound effect on these species. In contrast, the last glaciation did little to impede the ongoing population expansion of Geoffroy's cat (Fig. 5b), a habitat generalist that is made up of a large, single, unstructured population in Argentina and Uruguay, with expansions into Paraguay, Bolivia, and Brazil (Fernández et al. 2020). Our inferences of population expansion match two expansion events inferred from Bayesian Skyline analysis with mtDNA, dated 190-70 kya and 45-35 kya (Fernández et al. 2020). Consistent with their more restricted ranges in Chile, the Chilean pampas cat and guigna contain approximately twofold lower heterozygosity and higher ROH content than the Geoffroy's cat (Fig. 4d). The same pattern is found in other small carnivores east of the Andes such as Darwin's fox (*Lycalopex fulvipes*), the marine otter (*Lontra felina*) and southern river otter *(Lontra provocax*), all of which have small ranges, low levels of genetic diversity and elevated ROH content in the genome (Chavez et al. 2022; de Ferran et al. 2022). This contrasts with eastern counterparts, specifically the South American gray fox (*Lycalopex griseus*), a species that like the Geoffroy's cat occupies a large geographic range and possesses a high level of genetic diversity (Chavez et al. 2022). Together these studies demonstrate the major role of the Andes as a geographic barrier for small carnivore communities in southern South America.

### Conservation

Samples of the northern subspecies of guigna (*L. guigna tigrillo*), eastern tiger cat (*L. tigrinus emiliae*), Central American tiger cat (*L. tigrinus oncilla*) and margay from southern Brazil (*L. wiedii wiedii*) all contain a high fraction (>9%) of ROHs in their genome (Fig. 4d), similar to what is reported in fragile populations of Iberian lynx (Abascal et al. 2016). Together with low heterozygosity (guigna, eastern tiger cat) (Fig. 4d) and recent decline in *N_e_* (guigna, C. Am. tiger cat, margay) (Fig. 5b to d), these genomic measures are warning signs of an increasing vulnerability to issues mediated by low genetic diversity, such as inbreeding depression and a lack of adaptability to deal with a changing environment (Wilder et al. 2023). Each of these species is classified as Near Threatened or Vulnerable with a declining population trend by the IUCN Red List (Fig. 4d) and faces pressure from human-induced threats that may further lower population genetic diversity and long-term viability.

In sampled populations of two native forest habitat specialists, the northern subspecies of guigna and the margay population at the southern end of its range, population size has decreased in relation to anthropogenic deforestation and severe fragmentation of the respective Mediterranean forest in central Chile (Echeverría et al. 2006; Miranda et al. 2017) and Atlantic forest in southern Brazil (Ribeiro et al. 2009). Maintaining landscape connectivity will be crucial to avoid inbreeding and conserve genetic diversity, and small landscape elements such as remnant native forest fragments provide the vegetation cover necessary to act as corridors and safe havens within a mosaic of high agricultural pressure (Gálvez et al. 2013; García et al. 2020; Horn et al. 2020).

Given their distinct demographic histories since at least the LGM, Central American (*L. t. oncilla*) and Andean (*L. t. pardinoides*) populations of the clouded tiger cat should be managed as separate units for conservation. While the Andean population may be affected by various threats in parts of its range, including habitat loss, illegal trade, and retaliatory killing (Garrido and González-Maya 2011), the Central American population in particular should be considered as a conservation priority. It is isolated, not as genetically diverse as its Andean counterpart, and occupies a restricted range in a locality that is classified as under “significant concern” for conservation according to the IUCN World Heritage Outlook (IUCN World Heritage Outlook 2020). In our wild-caught eastern tiger cat sample, the observation of high ROH content is especially concerning, because it may translate into a compromised ability to fend off emerging pathogens (Sommer 2005), and disease transmission from free-ranging domestic dogs is a documented threat to this species (de Oliveira et al. 2020).

Unlike guigna and Iberian lynx and despite a comparably low genetic diversity, we did not recover indications of inbreeding or abrupt decline in *N_e_* in our sample of the endangered Andean cat, suggesting that the sampled population is currently stable. Conservation measures for this species should focus on the protection of suitable habitat (Lagos et al. 2020) and prey base (Napolitano et al. 2008), and mapping population connectivity to maintain natural levels of gene flow.

### Concluding Remarks

We obtained whole-genome sequencing data for a geographically and taxonomically broad sample of genus *Leopardus* and applied a number of reconstruction methods to obtain a robust phylogeny for this group, which includes novel placement of the Andean tiger cat and the exceedingly rare Andean cat. Phylogenomic discordance was found to result largely from extensive ILS, especially during the first pulse of speciation in the Pliocene, with additional contributions from historic introgression, in particular between the ocelot and the ancestral lineage to subgenus *Oncifelis*, and within the Pleistocene *Oncifelis* radiation. The large differences in genetic diversity, broadly consistent across three methods and two reference genomes, span the full spectrum of diversity levels found in the Felidae and reflect vastly different demographic histories that were likely shaped by the complex interactions between changes in climate and habitat/prey availability in the Neotropics, interspecies competition, population connectivity, and, in some cases, hybridization. As with other small carnivores, the southern Andes constitutes an important barrier that restricts the population size and genetic diversity of *Leopardus* populations in Chile. In summary, we improved our understanding of the most speciose extant felid radiation and demonstrated that carefully picking apart the contrasting genealogies found in whole-genome sequencing data can ultimately provide us with a more nuanced picture of a clade's evolutionary history.

## Materials and Methods

### Sampling and Sequencing

Throughout this study, we conservatively adhere to the taxonomic scheme of the IUCN cat specialist group (Kitchener et al. 2017), but see supplementary table S1, Supplementary Material online for the taxonomic details of each sample. We obtained whole-genome sequencing data of the 8 species of *Leopardus* recognized by this authority by combining existing data of 6 *Leopardus* samples available on the National Center for Biotechnology Information (NCBI) Sequence Read Archive (SRA) with new sequencing of 9 *Leopardus* samples. In addition, we included a puma (*Puma concolor*) and a jungle cat (*Felis chaus*) sample as outgroup species and an Iberian lynx (*Lynx pardinus*) and a leopard cat (*Prionailurus bengalensis*) sample as examples of wild cat species with unusually low or high heterozygosity, respectively (supplementary table S1, Supplementary Material online). We isolated high-quality DNA from frozen blood and tissue samples, taken opportunistically from road kills or animals in wildlife rescue centers. We did library preparation and sequenced whole-genome short reads on an Illumina HiSeq X platform. The genomes, with a size of ca. 2.4 Gbp, were sequenced at a 18 to 25 fold target depth (supplementary table S2, Supplementary Material online).

### Quality Control and Mapping

We used the SRA Toolkit v2.10.7 to convert downloaded data from the SRA Normalized Format to FASTQ format. To inspect the quality of the raw sequencing data of all samples, we used FastQC v0.11.9 (www.bioinformatics.babraham.ac.uk/projects/fastqc). FastQC reports were summarized with MultiQC v1.10.1 (Ewels et al. 2016). We excluded adapter sequences and low-quality reads with Trimmomatic v0.39 (Bolger et al. 2014) using the TruSeq3-PE standard library of adapters and filtering for an average read quality of 20 and minimum read lengths corresponding to the read lengths of the respective sequencing runs (supplementary table S2, Supplementary Material online).

We mapped the filtered reads to both the Canada lynx (*Lynx canadensis*) reference genome assembly (GenBank assembly accession GCA_007474595.2) produced as part of the Vertebrate Genome Project (Rhie et al. 2021) and the Geoffroy's cat (*Leopardus geoffroyi*) reference assembly (GenBank assembly accession GCA_018350155.1) (Bredemeyer et al. 2023), unpublished data). The Canada lynx is an outgroup species, equidistant to all samples in the focal genus, and therefore, an appropriate choice to avoid reference bias in our phylogenomic and introgression analyses. However, phylogenetic distance to the reference species is known to inflate estimates of heterozygosity and decrease the power to detect ROHs (Prasad et al. 2021). Therefore we rely primarily on the Geoffroy's cat reference, a congeneric ingroup species of close phylogenetic proximity to the other *Leopardus* samples, for analyses of individual genetic diversity and demography. Furthermore, replicating our analyses across both reference assemblies allows us to validate the consistency of our results.

Mapping was done with Minimap2 v2.17 (Li 2018) using the preset short read (sr) set of parameters. The sequence alignment files in BAM format were sorted and mate-related tags and MD tags were filled in with SAMtools v1.12 (Li et al. 2009). The BAM files were filtered for duplicate, unmapped, and low mapping quality (<10) reads, and nonchromosome level contigs were removed with SAMtools and GATK v3.8 (McKenna et al. 2010). To reduce storage needs, alignments were compressed with controlled loss of quality using Crumble v0.8.3 (Bonfield et al. 2019). We indexed the final BAM files with SAMtools and inspected the quality of the alignments with BamQC v0.1.25 (https://github.com/s-andrews/BamQC). Genome-wide, chromosome-level, and local sequencing depths after filtering and mapping were computed with deepTools v3.5.1 (Ramirez et al. 2016) and visually inspected with a custom R script (see Data and Code Availability).

### Base Calling

For use in downstream phylogenomic analyses, we called both reference and variant bases from the alignment files with ANGSD v0.933 (Korneliussen et al. 2014) to construct a pseudohaploid FASTA consensus genome for each sample. Bases were called randomly from the read stack, filtering for a minimum base quality of 20 and minimum mapping quality of 30. To further avoid calling from poorly aligned or pseudohomologous regions, we also filtered for site-specific sequencing depth where it deviated more than 50% from the average sequencing depth of a given chromosome for a given sample. Nucleotide frequencies and amount of missing data were counted with a custom Python script (see Data and Code Availability). To mask repetitive content in the genomic FASTA files, we first converted the RepeatMasker annotation output (www.repeatmasker.org) published with the reference genome (44% repetitive content) to the Browser Extensible Data (BED) format with BEDOPS v2.4.39 (Neph et al. 2012) and then used these coordinates to flag the repetitive regions as missing data (‘N’) in all samples with BEDtools v2.29.2 (Quinlan and Hall 2010). We split genomic FASTAs in nonoverlapping 100 kb GFs with BEDtools v2.29.2 (Quinlan and Hall 2010) and aligned all samples per fragment with custom Python scripts (see Data and Code Availability), removing fragments with >60% missing data for downstream analyses.

We also constructed a SNP data set in Variant Call Format (VCF) for analyses requiring SNP input. SNPs were called jointly for all samples from the alignment files with BCFtools v1.12 (Danecek et al. 2021) using the multiallelic caller method and filtering for a minimum sequencing depth of 5 and maximum depth of 40, a minimum base quality of 20, minimum mapping quality of 30 and excluding indels. For specific downstream analyses, we created a version of the call set were sex chromosomes were excluded and a version where repetitive content was masked with the reference genome RepeatMasker annotation, using the converted BED file in BCFtools. Quality and basic statistics of the SNP dataset, including heterozygosity, were assessed with the VariantQC module implemented in the DISCVR-seq Toolkit v1.3.9 (Yan et al. 2019).

### Mitogenome Assembly

To assemble complete mitochondrial genomes for each sample, we applied the MITObim pipeline (Hahn et al. 2013) using the mitogenome from the Canada lynx assembly (Rhie et al. 2021) as initial reference sequence to bait mitochondrial reads for each sample from the filtered FASTQ readpool. The procedure iteratively baits sequencing reads with the FASTA reference sequence, then maps fished reads to this reference and lastly derives a new reference sequence from the mapping assembly. The iterations are repeated until the mitogenome assembly cannot be improved any further. Base frequencies, amount of missing data, and the length of the final mitogenomic sequences were checked with a custom Python script (see Data and Code Availability). We aligned the final mitogenomes using MUSCLE v5.1 (Edgar 2004) with default parameters.

### Phylogeny

To infer the evolutionary relationships among *Leopardus* species, we constructed genomic phylogenies under the assumption of the MSC model. We judged the consistency of the results by using both NJ and ML approaches, multiple summary methods, and multiple iterations of the input data (with and without repetitive regions, with Canada lynx or Geoffroy's cat as reference genome). First, local NJ and ML phylogenetic trees were generated for each 100 kb GF. For the NJ approach, we calculated a pairwise distance matrix for each fragment using a custom Python script (see Code and Data Availability) and constructed a NJ tree for each matrix using the Python package scikit-bio v0.5.6 (https://scikit.bio). ML trees were computed with RAxML v8.2.12 (Stamatakis 2014) under de GTRGAMMA model of nucleotide evolution and default parameters, marking the puma sample as outgroup. For each fragment, the tree with the highest likelihood (‘bestTree’) was extracted as local ML tree.

We summarized the set of local NJ trees and the set of local ML trees separately, employing two different summary methods for each set: (1) a greedy consensus tree, and (2) an ASTRAL species tree. To produce a greedy consensus tree, the frequency of each clade (a monophyletic group defined by its constituent taxa) is counted in the set of local trees. A composite tree is then compiled with the clades that are most abundant in the tree set, displaying their frequency as nodal support value. As such, high nodal support indicates that many local trees in the genome contain the same grouping of taxa, while low support indicates a high amount of phylogenetic discordance. Unlike in a majority-rule consensus, where only nodes with >50% support are displayed, no such threshold is maintained in a greedy consensus. We computed the consensus with the program SumTrees v4.5.2 (https://github.com/jeetsukumaran/DendroPy), distributed with the DendroPy v4.5.2 Python library (Sukumaran and Holder 2010). SumTrees was run with the minimum clade frequency set to zero and otherwise default settings. The ASTRAL species tree was inferred with ASTRAL-III v5.7.1 (Zhang et al. 2018). From the set of input trees, the algorithm constructs the species tree that has the maximum number of shared induced quartet trees, a method that is statistically consistent under the MSC model. Based on the quartet scores, a local posterior probability (local PP) is computed for each node as measure of support. As an alternative to the summary of local trees for obtaining a consensus tree, we reduced the whole-genome alignment to a single genome-wide pairwise distance matrix by summing all the local pairwise distance matrices previously obtained for each fragment. We obtained a NJ tree from this matrix using the same method used for local NJ trees, and assessed robustness of the result by bootstrapping the set of local matrices. To enable comparison with previous phylogenetic studies in *Leopardus* that relied on mitochondrial data, we constructed a mitochondrial phylogeny from the aligned mitogenomes with RAxML v8.2.12 (Stamatakis 2014). RAxML was run with de GTRGAMMA model of nucleotide evolution and default parameters, using the puma as outgroup and 1000 bootstrap replicates to calculate node support.

### Divergence Time Estimation

To add a temporal context to the diversification of *Leopardus*, we estimated the time of divergence between each species. We first added an additional outgroup species, the jungle cat (*Felis chaus*), to improve the divergence time estimate of the root. The alignment was then pruned down to a single sample per species. For each 100 kb GF, we converted the alignment to the Phylogeny Inference Package (PHYLIP) format with a custom Python script (see Data and Code Availability) and recomputed the local ML tree with RAxML. We generated an MCMCtree control file for each GF, using the topology of its local ML tree with a custom Python script (see Data and Code Availability), and ran the MCMCtree program in PAML v4.9 (Yang 2007) with the alignment and control file as input to estimate the node ages of each local tree. We lack informative fossil data for the genus *Leopardus*, and therefore chose to constrain the root age with soft bounds between 14.04 and 6.83 mya, based on the minimum and maximum split times reported by Li et al. (2016) for the ocelot lineage with other lineages in Felinae. We used a global clock for sequence evolution, the Jukes and Cantor (1969) model of nucleotide substitution and a prior substitution rate of 2.26 × 10^−9^ substitutions per base pair per year, which is the estimated mutation rate in domestic cat (*Felis catus*) (Wang et al. 2022) and, when converted with a 3.8 yr generation time, is close to the 1 × 10^−8^ mutation rate per base pair per generation used in previous felid studies (Figueiró et al. 2017; Ramirez et al. 2022). For each GF, we sampled all parameters (sampfreq=1) generated after a burn-in of 500 generations during a run length of 2000 Markov chain generations, which were summarized in a single local tree. The resulting set of local trees in NEXUS format with node age estimates was summarized across the whole genome with SumTrees v4.5.2 (https://github.com/jeetsukumaran/DendroPy), with the minimum clade frequency set to zero, node age summary enabled and setting the edge lengths in the summary tree in accordance with the node ages. The age of each node is summarized by taking the mean of the node ages from the local trees that follow the consensus topology at the target node (i.e. the mean age of the most common clade at a given node). For each node age, a 95% Highest Posterior Density (HPD) interval was calculated.

We used MSMC2 v2.1.1 (Wang et al. 2020) to estimate the split time between recently diverged populations (<1 mya) with the rCCR. The rCCR is close to one when the within-population coalescent rates of both populations are highly similar and approaches zero as the populations diverge and their coalescent rates become dissimilar. The time point were the value of the rCCR drops down to half of its maximum value was taken to be the population split time (Schiffels and Wang 2020). We performed 20 bootstrap replicates and results were plotted using a custom Python script (see Data and Code Availability). We also used these coalescence rates to model an IM scenario with MSMC-IM (Wang et al. 2020). The model infers a cumulative migration probability backward through time, where the time point at 50% probability corresponds to the best estimate of the population split time. See section “Genomic diversity” for details on the MSMC2 methodology.

### Phylogenetic Discordance

To characterize the phylogenomic discordance in *Leopardus* genomes, we employed visual and quantitative methods to the set of local ML trees. Using the Newick utilities v1.6 (Junier and Zdobnov 2010), we counted the total amount of unique topologies as well as the relative frequencies of the most frequent topologies. To visualize the discordance in trees retrieved along the genome, we made an overlay of all local trees using DensiTree v2.01 (Bouckaert 2010). For a clearer view of only the most persistent alternative edges in the complete tree set, local trees were summarized as an implicit network. We randomly subsampled 20% of the tree set as input to the Consensus Network algorithm in SplitsTree5 (Huson and Bryant 2006), using median edge weights and a minimum threshold of 20% for inclusion of edges. We further quantified genome-wide discordance by projecting node-specific discordance on each internal node of the consensus tree with PhyParts v0.0.1 (Smith et al. 2015). PhyParts summarizes conflict in a tree set by counting the frequency of the target node as it appears in the species tree, the most common alternative topology, and the total of all other alternative splits. These are plotted as pie charts on every node with a Python script (https://github.com/mossmatters/MJPythonNotebooks). In addition to the internal nodes, we assessed the stability of every sample's position within the tree set by calculating the LSI using Phyutility v2.2.6 (Smith and Dunn 2008). The index is calculated as the average of the frequencies with which each rooted triplet subtree containing the taxon is found within the tree set (Thorley and Wilkinson 1999). For each sample, we also counted its frequency of attachment to all other branches in the consensus throughout the tree set with the Branch Attachment Frequency (BAF) function developed in Phyutility (Smith and Dunn 2008).

### Introgression

To distinguish between ILS and introgressive hybridization as drivers of topological discordance among local trees, we conducted three separate tests for introgression that each leverage a distinct aspect of the data. Based on imbalances in derived mutations shared among non-sister species counted as ABBA/BABA site patterns (Green et al. 2010), we computed genome-wide D-statistics with Dsuite v0.4 (Malinsky et al. 2021). This program allows for systematic, genome-scale calculation of D- and related statistics from VCF files, accounting for all possible triplet topologies and a fixed outgroup. With our masked SNP dataset as input, we computed Patterson's D and the *f*_4_ admixture ratio (Patterson et al. 2012) for all triplets included in the greedy consensus tree, using the Dtrios function with default settings. A default amount of 20 jackknife blocks was used to calculate statistical support for the D-statistics. The results were plotted as heatmaps with Ruby scripts (plot_d.rb and plot_f4ratio.rb) made available by the developers of Dsuite. To address the correlation of D- and *f*-statistics among taxa that share an internal or terminal branch in the triplet analysis, Dsuite includes the *f*-branch statistic (Malinsky et al. 2018). This statistic summarizes introgression signals in taxa that share a clade with either the donor or receiver taxon involved in the actual introgression event. It does so by accounting for the *f*_4_ admixture ratio specific to internal and terminal branches of the species tree. We calculated and plotted the *f*-branch statistic with Dsuite using the previously obtained *f*_4_ admixture ratios as input.

Our second approach took advantage of the information contained in the branch lengths of our tree set by applying the Quantifying Introgression via Branch Lengths (QuIBL) program (Edelman et al. 2019) available on GitHub (https://github.com/miriammiyagi/QuIBL). This test functions independently from tests based on topological imbalances because it relies on the branch lengths, not topologies, of local trees to segregate introgression from ILS. QuIBL compares the internal branch lengths of all relevant 3-taxon subtrees (triplets) of a given local tree to the genome-wide distribution of branch lengths in those triplets to score the likelihood that the local tree reflects introgression. We randomly subsampled our local tree set to 20% of the trees for computational feasibility and ran QuIBL with default settings, except for an increase in the number of total expectation-maximization steps from 10 to 50, as recommended for thousands of trees. The output of this analysis, a table with distribution models and associated estimates for each possible triplet, was processed and plotted in R with the “quiblR” package (https://github.com/nbedelman/quiblR). We organized the plot according to the topology of the greedy consensus tree.

As a third approach, we examined topological imbalances in local trees by constructing an explicit phylogenetic network from the tree set with the Species Networks applying Quartets (SNaQ) method (Solís-Lemus and Ané 2016) implemented in the Julia package PhyloNetworks v0.14 (Solís-Lemus et al. 2017), using a custom Julia script (see Data and Code Availability). As input, SNaQ uses information about the proportion of local topologies that support a given node in the species tree, as well as proportions in support of alternative nodes, expressed as node-specific CF. The CFs are calculated from all possible 4-taxon subtrees (quartets) and allow distinction between ILS and introgression as a cat and pampasmost likely explanation for a reticulate branch. A maximum pseudolikelihood computation evaluates all the CFs and infers the optimal phylogenetic network. From the set of input trees, we estimated concordance factors with credibility intervals and proceeded with the SNaQ analysis, which is conducted as a sequence of searches. The first search infers the optimal network without reticulate branches (h0), and all subsequent searches start from the previous optimal network while allowing for one additional reticulation, up to three (h1-h3). To find the optimal network for a given amount of reticulations, we iterated the search 50 times. The most appropriate number of reticulations is then determined by inspecting the slope of the log-pseudolikelihood of each optimal network as it increases with number of reticulations, choosing the network for which a further increase in reticulations does not lead to a substantial increase in log-pseudolikelihood.

### Genomic Diversity

To examine the amount of genomic diversity within and among *Leopardus* species, we estimated genetic structure and nucleotide divergence among samples, and heterozygosity and ROHs for each sample. We assessed structure among samples with a genomic PCA of the SNP data set using PLINK v2.0 (Purcell et al. 2007). A separate PCA was conducted for (i) all samples excl. the outgroup, (ii) subgenera “*Leopardus*” and “*Lynchailurus*”, i.e. ocelot, margay, Andean cat, and pampas cat, and (iii) subgenus “*Oncifelis*”. To account for linkage disequilibrium, we pruned SNPs that had a squared correlation greater than 0.1 when considered pairwise to other SNPs in a 50-SNP window. SNPs with a minor allele frequency below 0.05 were excluded. We extracted up to 10 principal components and plotted eigenvectors with a custom Python script (see Data and Code Availability).

We calculated a genome-wide pairwise distance matrix from the pseudohaploid consensus genomes using a custom Python script (as in the section “Phylogeny”) to evaluate the diversity between samples. Percentage pairwise distance was considered as nucleotide diversity (*π*) when comparing samples of the same species and as nucleotide divergence ($D_{xy}$) when comparing samples of different species. In addition, pairwise mitochondrial sequence divergence was estimated from the mitogenomes using the default setting in MEGA7 (Kumar et al. 2016).

To evaluate the genomic diversity within each sample, we estimated autosomal rates of heterozygosity and identified ROHs under a Bayesian approach implemented in ROHan v1.0 (https://github.com/grenaud/rohan). ROHan uses the filtered BAM file of each individual sample as input and proceeds in three steps. It uses genotype likelihoods to estimate the average heterozygosity. Using that information, ROHs are identified throughout the genome. Heterozygosity is then re-estimated for the proportion of the genome within ROHs and for the genome excluding ROHs. As prior for the expected heterozygosity within ROHs (−rohmu), we used 0.0002 differences per base pair. Heterozygosity and PP of identified ROHs were plotted for each sample along all chromosomes using ROHan. We validated our estimates of heterozygosity with two other methods. We calculated the site allele frequency likelihood from the BAM files with ANGSD v0.921 (Korneliussen et al. 2014), based on individual genotype likelihoods using the SAMtools algorithm and filtering for reads with a minimum mapping quality of 30 and bases with a minimum quality of 20. To obtain the proportion of heterozygous sites from the site allele frequency likelihoods, we computed the folded site frequency spectrum for each sample using the realSFS functionality of ANGSD and a custom Python script (see Data and Code Availability). The computation was done in 200 kb nonoverlapping fragments to obtain a measure of the genome-wide variation in local heterozygosity. We then calculated the mean value and interquartile range of the local estimates using a custom R script (see Data and Code Availability). Finally, we retrieved the number of heterozygous sites in each sample in the SNP data set from the VariantQC reports generated during quality control, and obtained the relative heterozygosity by dividing the number of heterozygous sites by the total number of sites, including non-variants, that were called in the VCF pipeline (see section “Base Calling”).

We inferred population sizes through time with MSMC2 v2.1.1 (Wang et al. 2020) to gain insight in the demographic history of each *Leopardus* species. The effective sequencing depth of all samples mapped to Geoffroy's cat (17 to 27×, see supplementary table S4, Supplementary Material online) was close to or above the 18× recommended for sequentially Markovian coalescent methods (Nadachowska-Brzyska et al. 2016). We generated a mappability mask specific to the Geoffroy's cat reference genome with SNPable Regions (http://lh3lh3.users.sourceforge.net/snpable.shtml), to determine the regions on the chromosomes on which short sequencing reads can be uniquely mapped. Variants were called from the BAM files for all autosomes with SAMtools and the bamCaller.py script from the MSMC Tools repository (https://github.com/stschiff/msmc-tools). The same filters were applied as described previously for the SNP data set, but we called each sample separately and masked regions of the genome with the mappability mask and where the sequencing depth was either above twice the chromosome-level average depth or below half this depth. We prepared the input files required for MSMC2 with the generate_multihetsep.py script from the MSMC Tools repository and ran MSMC2 on each sample separately (i.e. two haplotypes in each run) with default settings, but reducing the number of free parameters in the demographic model from 28 to 23, in a pattern of 27 fixed time segments instead of 32 (-p 1*2 + 20*1 + 1*2 + 1*3). The reduction in free parameters should partly mitigate the effects of model overfitting, often manifested as extreme estimates of the effective population size in the most recent or most ancient time intervals (Schiffels and Wang 2020). We assumed a mutation rate of 0.86 × 10^−8^ mutations per site per generation and a generation time of 3.8 yr as in domestic cat (*Felis catus*) (Wang et al. 2022) to scale the time in coalescent units to years. For each sample, 20 bootstrap replicates were performed with the multihetsep_bootstrap.py script included in the MSMC Tools repository. Consensus estimates of effective population sizes were calculated from the bootstrap replicates and plotted with a custom Python script (see Data and Code Availability).

## Supplementary Material

msad255_Supplementary_Data

## Data Availability

All sequencing data have been archived in the SRA of the NCBI under the BioProject accession number PRJNA985552. Mitogenome assemblies have been archived in NCBI BankIt under accessions OR257581, OR257582, OR556000, and OR822031-OR822035.

## Code Availability

To achieve high reproducibility, large parts of the workflow were implemented in the Snakemake v5.3.0 workflow management system (Mölder et al. 2021) and executed within controlled software environments deployed with the Conda package management system (https://conda.io). Snakemake code, details about the associated software environments and miscellaneous scripts are available on an archived GitHub repository (https://github.com/jlescroart/Lescroart2023). The Snakemake code covers six interlinked pipelines: (i) quality control and mapping of raw reads (‘fastq2bam’), (ii) assemblage of mitogenomes from raw reads and subsequent phylogenetic analysis (‘fastq2mtdna’), (iii and iv) base calling into consensus genomes and a SNP data set (resp. “bam2fasta” and “bam2vcf”), (v) phylogenomic analysis (‘phylogeny’), and (iv) genomic diversity measures (‘diversity’).
