# Correction to: Extensive Phylogenomic Discordance and the Complex Evolutionary History of the Neotropical Cat Genus *Leopardus*

**DOI:** 10.1093/molbev/msae005

**Published:** 2024-02-02

This is a correction to: Jonas Lescroart, Alejandra Bonilla-Sánchez, Constanza Napolitano, Diana L Buitrago-Torres, Héctor E Ramírez-Chaves, Paola Pulido-Santacruz, William J Murphy, Hannes Svardal, Eduardo Eizirik, Extensive Phylogenomic Discordance and the Complex Evolutionary History of the Neotropical Cat Genus *Leopardus*, *Molecular Biology and Evolution*, Volume 40, Issue 12, December 2023, msad255, https://doi.org/10.1093/molbev/msad255

The following changes have been made to the original publication of this manuscript. The affiliation of third author Constanza Napolitano has been changed in translation to now read: “Departamento de Ciencias Biológicas y Biodiversidad, Universidad de Los Lagos, Osorno, Chile”.

In **Acknowledgments** section, first part of the second sentence now reads: “C.N. was supported by Agencia Nacional de Investigación y Desarrollo[…]” instead of: “C.L. was supported by Agencia Nacional de Investigación y Desarrollo[…]”. The end of the sixth sentence now reads: “[…], the Mammal Collection of the Regional University of Blumenau, Brazil, and the Tissue Collection of the Alexander von Humboldt Institute, Colombia, for valuable support in sample collection.” instead of: “[…]and the Mammal Collection of the Regional University of Blumenau, Brazil for valuable support in sample collection.”

These emendations are made in the article.

