# Supplementary material for: Extensive Phylogenomic Discordance and the Complex Evolutionary History of the Neotropical Cat Genus Leopardus
Source: Mol Biol Evol. 2023 Nov 21;40(12):msad255. doi: 10.1093/molbev/msad255 (PMC10701098; doi:10.1093/molbev/msad255)

SUPPLEMENTARY MATERIALS

# Extensive phylogenomic discordance and the complex evolutionary history of the Neotropical cat genus *Leopardus*

Jonas Lescroart, Alejandra Bonilla-Sánchez, Constanza Napolitano, Diana L. Buitrago-Torres, Héctor E. Ramírez-Chaves, Paola Pulido-Santacruz, William J. Murphy, Hannes Svardal, Eduardo Eizirik

Supplementary materials contain:

- supplementary text;
- 10 supplementary tables;
- 17 supplementary figures.

1 SUPPLEMENTARY TEXT

1.1 Mapping to Geoffroy's cat reference assembly

We mapped filtered reads of each sample to the Geoffroy's cat (*Leopardus geoffroyi*) reference assembly (GenBank assembly accession GCA_018350155.1, Bredemeyer et al. *in prep*). Mapping, base calling and generating a set of GFs proceeded as described for the Canada lynx reference genome (see Materials and Methods). The resulting effective sequencing depths varied between 17-27× (excl. the outgroup sample at 30×). We called bases from the BAM files for each sample to retrieve pseudohaploid consensus genomes, which we masked for the 34% of repetitive content found in the reference genome. The remaining bases in the consensus genomes covered 83-95% of the non-repetitive part of the reference (Table S4). These genomic FASTAs were split into 24,233 GFs with a length of 100kb. A set of 21,294 GFs (88%) remained after removing all alignments in which at least one sample had more than 60% missing data. In addition to GFs from consensus genomes, we also created a variant call set from the BAM files, calling 3-9 million SNPs per sample, excluding the outgroup samples with 17-21 million SNPs (Table S4). After masking regions with repetitive content in the reference genome, 2-6 million SNPs remained per ingroup sample.

1.2 Consensus genomes not masked for repetitive content

To evaluate the consistency of phylogenomic results with and without inclusion of repetitive genomic regions, we redid our phylogenetic analyses on unmasked versions of the pseudohaploid consensus genomes, using the same methodology as described in the Materials and Methods. Between 81-91% of the reference genome was covered by the unmasked consensus genomes with the Canada lynx reference and 81-95% with the Geoffroy's cat reference. The genomic FASTAs were split in 100kb GFs, with 23,007 GFs (96%) and 23,252 GFs (96%) remaining for the respective references after removing all alignments in which at least one sample had more than 60% missing data. Across phylogenetic results derived from both reference genomes, all but one (ASTRAL using ML tree set with

Canada lynx reference) of the summary methods applied to unmasked data supported the topology shown in Figure 2A.

### 1.3 Demographic history of sample 'Pampas cat (Zoo)'

WGS data of the sample 'Pampas cat (Zoo)', as for other samples used in this study sequenced by Li et al. (2019), was sequenced from a cell culture derived from an individual in captivity with unknown provenance. For that reason, until more pampas cat samples with documented morphology and geographic origin become available for sequencing, we cannot allocate the current sample to any of the proposed clades in the pampas cat species complex (Kitchener et al. 2017, Nascimento et al. 2020). The demographic history of our pampas cat sample from Chile (Figure 5), which represents the Central Chilean pampas cat *Leopardus colocola colocola* according to Kitchener et al. (2017), is very different from that of the captive sample (Figure S17), indicating that each pampas cat group conceivably has its own distinct demographic history. Therefore, we cannot currently know which group is represented by the demography inferred from sample 'Pampas cat (Zoo)'. In addition, its demographic trajectory shows a large and abrupt increase, then decrease in equal measure, in effective population size ($N_e$) within the most recent estimates. This result could be an artefact of potential out- and inbreeding during breeding programs in captivity, a conclusion partially support by the high fraction of ROHs in the sample. A similar spike in estimates of recent $N_e$ was observed in a puma (*Puma concolor*) sample with mixed ancestry followed by close inbreeding (Saremi et al. 2019) and in samples of captive lions (*Panthera leo*) (Armstrong et al. 2020).

### 1.4 References

Abascal, F., A. Corvelo, F. Cruz, J. L. Villanueva-Canas, A. Vlasova, M. Marcet-Houben, B. Martinez-Cruz, J. Y. Cheng, P. Prieto, V. Quesada, J. Quilez, G. Li, F. Garcia, M. Rubio-Camarillo, L. Frias, P. Ribeca, S. Capella-Gutierrez, J. M. Rodriguez, F. Camara, E. Lowy, L. Cozzuto, I. Erb, M. L. Tress, J. L. Rodriguez-Ales, J. Ruiz-Orera, F. Reverter, M. Casas-Marce, L. Soriano, J. R. Arango, S. Derdak, B. Galan, J. Blanc, M. Gut, B. Lorente-Galdos, M. Andres-Nieto, C. Lopez-Otin, A. Valencia, I. Gut, J. L. Garcia, R. Guigo, W. J. Murphy, A. Ruiz-Herrera, T. Marques-Bonet, G. Roma, C. Notredame, T. Mailund, M. M. Alba, T. Gabaldon, T. Alioto, and J. A. Godoy. 2016. Extreme genomic erosion after recurrent demographic bottlenecks in the highly endangered Iberian lynx. Genome Biology **17**.

Armstrong, E. E., R. W. Taylor, D. E. Miller, C. B. Kaelin, G. S. Barsh, E. A. Hadly, and D. Petrov. 2020. Long live the king: chromosome-level assembly of the lion (Panthera leo) using linked-read, Hi-C, and long-read data. BMC Biology **18**:3.
Babraham Bioinformatics. 2005. FastQC.
Babraham Bioinformatics. 2014. BamQC.
Edelman, N. B., P. B. Frandsen, M. Miyagi, B. Clavijo, J. Davey, R. B. Dikow, G. Garcia-Accinelli, S. M. Van Belleghem, N. Patterson, D. E. Neafsey, R. Challis, S. Kumar, G. R. P. Moreira, C. Salazar, M. Chouteau, B. A. Counterman, R. Papa, M. Blaxter, R. D. Reed, K. K. Dasmahapatra, M. Kronforst, M. Joron, C. D. Jiggins, W. O. McMillan, F. Di Palma, A. J. Blumberg, J. Wakeley, D. Jaffe, and J. Mallet. 2019. Genomic architecture and introgression shape a butterfly radiation. Science **366**:594-+.
Huson, D. H., and D. Bryant. 2006. Application of phylogenetic networks in evolutionary studies. Molecular Biology and Evolution **23**:254-267.
Kitchener, A. C., C. Breitenmoser-Würsten, E. Eizirik, A. Gentry, L. Werdelin, A. Wilting, N. Yamaguchi, and W. E. Johnson. 2017. A revised taxonomy of the Felidae: The final report of the Cat Classification Task Force of the IUCN/SSC Cat Specialist Group. Cat News.
Korneliussen, T. S., A. Albrechtsen, and R. Nielsen. 2014. ANGSD: Analysis of Next Generation Sequencing Data. Bmc Bioinformatics **15**.
Kumar, S., G. Stecher, and K. Tamura. 2016. MEGA7: Molecular Evolutionary Genetics Analysis Version 7.0 for Bigger Datasets. Molecular Biology and Evolution **33**:1870-1874.
Li, G., B. W. Davis, E. Eizirik, and W. J. Murphy. 2016. Phylogenomic evidence for ancient hybridization in the genomes of living cats (Felidae). Genome Research **26**:1-11.
Li, G., H. V. Figueiró, E. Eizirik, and W. J. Murphy. 2019. Recombination-Aware Phylogenomics Reveals the Structured Genomic Landscape of Hybridizing Cat Species. Molecular Biology and Evolution.
Nascimento, F. O. D., J. Cheng, and A. Feijó. 2020. Taxonomic revision of the pampas cat Leopardus colocola complex (Carnivora: Felidae): an integrative approach. Zoological Journal of the Linnean Society.
Nascimento, F. O. D., and A. Feijó. 2017. Taxonomic revision of the tigrina Leopardus tigrinus (Schreber, 1775) species group (Carnivora, Felidae). Papéis Avulsos de Zoologia **57**:231-264.
Ramirez, F., D. P. Ryan, B. Gruning, V. Bhardwaj, F. Kilpert, A. S. Richter, S. Heyne, F. Dundar, and T. Manke. 2016. deepTools2: a next generation web server for deep-sequencing data analysis. Nucleic Acids Research **44**:W160-W165.
Ramirez, J. L., J. Lescroart, H. V. Figueiró, J. P. Torres-Florez, P. M. S. Villela, L. L. Coutinho, P. D. Freitas, W. E. Johnson, A. Antunes, P. M. Galetti Jr., and E. Eizirik. 2022. Genomic Signatures of Divergent Ecological Strategies in a Recent Radiation of Neotropical Wild Cats. Molecular Biology and Evolution **39**.
Renaud, G. 2019. ROHan: inference of heterozygosity rates and runs of homozygosity for modern and ancient samples. GitHub.
Saremi, N. F., M. A. Supple, A. Byrne, J. A. Cahill, L. L. Coutinho, L. Dalén, H. V. Figueiró, W. E. Johnson, H. J. Milne, S. J. O'Brien, B. O'Connell, D. P. Onorato, S. P. D. Riley, J. A. Sikich, D. R. Stahler, P. M. S. Villela, C. Vollmers, R. K. Wayne, E. Eizirik, R. B. Corbett-Detig, R. E. Green, C. C. Wilmers, and B. Shapiro. 2019. Puma genomes from North and South America provide insights into the genomic consequences of inbreeding. Nature Communications **10**:4769.
Sartor, C. C., S. A. Cushman, H. Y. Wan, R. Kretschmer, J. A. Pereira, N. Bou, M. Cosse, S. González, E. Eizirik, T. R. O. de Freitas, and T. C. Trigo. 2021. The role of the environment in the spatial dynamics of an extensive hybrid zone between two neotropical cats. Journal of Evolutionary Biology **34**:614-627.
Solís-Lemus, C., P. Bastide, and C. Ané. 2017. PhyloNetworks: A Package for Phylogenetic Networks. Molecular Biology and Evolution **34**:3292-3298.
Wang, K., I. Mathieson, J. O'Connell, and S. Schiffels. 2020. Tracking human population structure through time from whole genome sequences. Plos Genetics **16**.

Wang, R. J., M. Raveendran, R. A. Harris, W. J. Murphy, L. A. Lyons, J. Rogers, and M. W. Hahn. 2022. De novo Mutations in Domestic Cat are Consistent with an Effect of Reproductive Longevity on Both the Rate and Spectrum of Mutations. Molecular Biology and Evolution **39**:msac147.

Yan, M. Y., B. Ferguson, and B. N. Bimber. 2019. VariantQC: a visual quality control report for variant evaluation. Bioinformatics **35**:5370-5371.

2 SUPPLEMENTARY TABLES

Table S1: Samples used in this study

| Sample ID | Alternative sample IDs | (Sub)species *sensu* Kitchener et al. (2017)* | Sex | Geographic origin | NCBI SRA Experiment | Sample source/supplier |
|---|---|---|---|---|---|---|
| Ocelot (Zoo) | LPA-6 | *Leopardus pardalis* | ♂ | Unkown (captive) | SRX3213484 | Li et al. (2019) |
| Ocelot (USA) | wmLPA-1 | *Leopardus pardalis pardalis* | ♀ | Texas, USA | SRX20812397 | This study (W.J.M.) |
| Ocelot (Brazil) | LP339 | *Leopardus pardalis mitis* | ♂ | São Paulo, Brazil | SRX15135580 | Ramirez et al. (2022) |
| Margay | 398-wiedii | *Leopardus wiedii wiedii* | ♀ | Rio Grande do Sul, Brazil | SRX15135581 | Ramirez et al. (2022) |
| Andean cat | LJA-1 | *Leopardus jacobita* | ♂ | La Paz, Bolivia | SRX20812398 | This study (C.N.) |
| Pampas cat (Zoo) | LCO-3 | *Leopardus colocola* | ♂ | Unkown (captive) | SRX3213485 | Li et al. (2019) |
| Pampas cat (Chile) | Lcol1CL | *Leopardus colocola colocola* | ♀ | Valparaíso Region, Chile | SRX20812399 | This study (C.N.) |
| Guigna | Lgui1CL | *Leopardus guigna tigrillo* | ♂ | O'Higgins Region, Chile | SRX20812400 | This study (C.N.) |
| Geoffroy’s cat | OGE-3 | *Leopardus geoffroyi* | ♂ | Unkown (captive) | SRX3213482 | Li et al. (2019) |
| Southern tiger cat | GU_18963; GU115; FURB18963; bLgt652 | *Leopardus guttulus* | ♂ | Santa Catarina, Brazil | SRX20812401 | Sartor et al. (2021); this study: WGS sequencing |
| C. Am. tiger cat | LTI-13 | *Leopardus tigrinus oncilla* | ♂ | Costa Rica | SRX20812402 | Li et al. (2016); this study: WGS sequencing |
| Andean tiger cat 32451 | LTI-32451 | *Leopardus tigrinus pardinoides* | ♀ | Antioquia, Colombia | SRX20812403 | This study (A.B.S.) |
| Andean tiger cat 32454 | LTI-32454 | *Leopardus tigrinus pardinoides* | ♀ | Caldas, Colombia | SRX20812404 | This study (D.L.B.T. & H.R.C.) |
| Eastern tiger cat (Zoo) | LTI-6 | *Leopardus tigrinus emiliae* | ♂ | Unkown (captive) | SRX3213483 | Li et al. (2019) |
| Eastern tiger cat (Brazil) | LTI-699, bLti242 | *Leopardus tigrinus emiliae* | ♂ | Ceará, Brasil | SRX20812405 | This study (L. Teixeira) |
| Puma | SC36_Marlon | *Puma concolor* | ♂ | California, USA | SRX4067834-SRX4067840 | Saremi et al. (2019) |
| Jungle cat | Fch-1a | *Felis chaus* | ♂ | Unkown (captive) | SRX9766946 | K. Bredemeyer (2020) |
| Iberian lynx | LYNX9; A010; Borja | *Lynx pardinus* | ♂ | Andalucía, Spain | ERX1327192 | Abascal et al. (2016) |
| Leopard cat | PBE_2350 | *Prionailurus bengalensis* | ♂ | Unkown (captive) | SRX8880503-SRX8880504 | Saint-Petersburg State University (2021) |

* In this study we conservatively adhere to the taxonomic scheme published by the IUCN cat specialist group (Kitchener et al. 2017) with the exception of including the subspecies eastern tiger cat (*L. tigrinus emiliae*), a choice that was made to distinguish this northeast Brazilian population of northern tiger cat (*L. tigrinus*) from the species' unsampled type population in French Guiana (*L. tigrinus tigrinus*). It should be noted that, based on morphological, ecological and limited genetic evidence, the eastern tiger cat has been proposed as a full species (*L. emiliae*) (Nascimento and Feijó 2017) and five distinct species have been proposed for the pampas cat species complex (Nascimento et al. 2020). The latter is a closely related, monophyletic group represented in our study by two samples belonging to one or two of these five taxa.

Table S2: Sequencing details

| **Sample ID** | **Platform** | **Read length** | **Number of raw reads** | **Raw read sequencing depth (×)** | **Duplicate reads (%)** | **GC content (%)** | **≥Q30 (%)** |
|---|---|---|---|---|---|---|---|
| Ocelot (Zoo) | Illumina HiSeq 2000 | 2 x 125 | 539,846,424 | 28 | 24 | 40 | 87 |
| Ocelot (USA) | Illumina HiSeq X Ten | 2 x 150 | 483,882,702 | 30 | 7 | 42 | 91 |
| Ocelot (Brazil) | Illumina HiSeq 2500 | 2 x 100 | 473,771,504 | 20 | 17 | 42 | 93 |
| Margay | Illumina HiSeq 2500 | 2 x 100 | 463,619,760 | 19 | 17 | 42 | 89 |
| Andean cat | Illumina HiSeq X Ten | 2 x 150 | 444,026,656 | 27 | 7 | 41 | 95 |
| Pampas cat (Zoo) | Illumina HiSeq 2000 | 2 x 125 | 587,225,672 | 30 | 12 | 40 | 85 |
| Pampas cat (Chile) | Illumina HiSeq | 2 x 150 | 379,965,930 | 23 | 11 | 44 | 89 |
| Guigna | Illumina HiSeq | 2 x 150 | 377,278,448 | 23 | 8 | 43 | 91 |
| Geoffroy's cat | Illumina HiSeq 2000 | 2 x 125 | 629,309,440 | 32 | 14 | 40 | 86 |
| Southern tiger cat | Illumina HiSeq X Ten | 2 x 150 | 315,337,198 | 19 | 7 | 41 | 88 |
| C. Am. tiger cat | Illumina HiSeq X Ten | 2 x 150 | 489,727,164 | 30 | 8 | 42 | 91 |
| Andean tiger cat 32451 | Illumina HiSeq X Ten | 2 x 150 | 315,109,264 | 19 | 6 | 43 | 94 |
| Andean tiger cat 32454 | Illumina HiSeq X Ten | 2 x 150 | 319,777,098 | 20 | 7 | 42 | 94 |
| Eastern tiger cat (Zoo) | Illumina HiSeq 2000 | 2 x 125 | 807,739,352 | 42 | 33 | 40 | 86 |
| Eastern tiger cat (Brazil) | Illumina HiSeq X Ten | 2 x 150 | 438,357,926 | 27 | 7 | 42 | 95 |
| Puma | Illumina HiSeq 2500,<br>Illumina HiSeq 4000 | 2 x 100,<br>2 x 150 | 1,012,495,705 (100bp)<br>292,384,968 (150bp) | 60 | 9 | 41 | 85 |
| Jungle cat | Illumina NovaSeq 6000 | 2 x 150 | 557,273,742 | 35 | 19 | 41 | 96 |
| Iberian lynx | Illumina HiSeq 2000 | 2 x 100 | 708,079,508 | 30 | 21 | 39 | 83 |
| Leopard cat | Illumina HiSeq 2500 | 2 x 125 | 1,027,952,524 | 54 | 10 | 42 | 88 |

Raw read sequencing depth was calculated as (read length x number of raw reads)/length of the reference genome. Number of duplicate reads, total GC content and the Q30 statistic were calculated with FastQC (Babraham Bioinformatics 2005).

Table S3: Mapping to Canada lynx (*Lynx canadensis*) reference genome (mLynCan4.pri.v2) and base calling details

| Sample ID | Reads after filtering and mapping (%) | Effective sequencing depth (×) | Coverage of ref. genome (%) | Coverage of non-repetitive part of the ref. genome (%) | SNPs |
|---|---|---|---|---|---|
| Ocelot (Zoo) | 65 | 18 | 86 | 89 | 16,764,306 |
| Ocelot (USA) | 87 | 26 | 91 | 95 | 16,966,161 |
| Ocelot (Brazil) | 89 | 18 | 81 | 84 | 16,760,515 |
| Margay | 87 | 17 | 79 | 82 | 15,996,278 |
| Andean cat | 89 | 25 | 90 | 95 | 15,483,429 |
| Pampas cat (Zoo) | 71 | 22 | 81 | 86 | 16,100,088 |
| Pampas cat (Chile) | 83 | 20 | 81 | 84 | 15,794,103 |
| Guigna | 87 | 21 | 87 | 91 | 15,465,501 |
| Geoffroy’s cat | 69 | 23 | 81 | 86 | 16,092,828 |
| Southern tiger cat | 85 | 17 | 87 | 91 | 16,228,541 |
| C. Am. tiger cat | 87 | 27 | 90 | 95 | 15,717,930 |
| Andean tiger cat 32451 | 86 | 17 | 89 | 93 | 16,075,782 |
| Andean tiger cat 32454 | 87 | 18 | 88 | 93 | 16,071,274 |
| Eastern tiger cat (Zoo) | 54 | 23 | 85 | 89 | 15,642,646 |
| Eastern tiger cat (Brazil) | 89 | 25 | 91 | 95 | 15,573,139 |
| Puma | 56 | 34 | 83 | 88 | 15,189,941 |
| Jungle cat | 57 | 20 | - | 77 | - |
| Iberian lynx | 72 | 21 | - | - | 2,800,102 |
| Leopard cat | 81 | 44 | - | - | 16,657,223 |

The number of reads retained in the filtered BAM files was counted with BamQC (Babraham Bioinformatics 2014) and divided by the initial number of raw reads to obtain the percentage. The effective sequencing depth was estimated from the filtered BAMs with deepTools (Ramirez et al. 2016). Coverage refers to the percentage of successfully called bases for sites that are present in the reference sequence and is shown for the total reference genome and the non-repetitive (repeatmasked) part of the reference. The number of SNPs per sample in the unmasked SNP set was counted with VariantQC (Yan et al. 2019).

Table S4: Mapping to Geoffroy's cat (*Leopardus geoffroyi*) reference genome (O.geoffroyi_Oge1_pat1.0) and base calling details

| **Sample ID** | **Reads after filtering and mapping (%)** | **Effective sequencing depth (×)** | **Coverage of ref. genome (%)** | **Coverage of non-repetitive part of the ref. genome (%)** | **SNPs** |
|---|---|---|---|---|---|
| Ocelot (Zoo) | 66 | 19 | 88 | 89 | 8,364,001 |
| Ocelot (USA) | 89 | 27 | 94 | 95 | 8,570,210 |
| Ocelot (Brazil) | 92 | 18 | 85 | 86 | 8,399,981 |
| Margay | 91 | 18 | 83 | 83 | 7,840,257 |
| Andean cat | 91 | 25 | 93 | 95 | 7,180,043 |
| Pampas cat (Zoo) | 74 | 23 | 87 | 88 | 7,688,242 |
| Pampas cat (Chile) | 84 | 20 | 84 | 85 | 7,303,651 |
| Guigna | 89 | 21 | 91 | 92 | 3,340,889 |
| Geoffroy's cat | 74 | 24 | 90 | 91 | 2,809,617 |
| Southern tiger cat | 87 | 17 | 92 | 92 | 3,995,353 |
| C. Am. tiger cat | 88 | 27 | 84 | 95 | 4,201,388 |
| Andean tiger cat 32451 | 88 | 17 | 92 | 94 | 4,610,484 |
| Andean tiger cat 32454 | 89 | 18 | 93 | 94 | 4,603,420 |
| Eastern tiger cat (Zoo) | 55 | 23 | 90 | 91 | 3,786,278 |
| Eastern tiger cat (Brazil) | 91 | 25 | 95 | 95 | 3,698,457 |
| Puma | 56 | 30 | 81 | 83 | 18,961,201 |
| Jungle cat | 57 | 20 | - | - | - |
| Iberian lynx | 70 | 21 | - | - | 17,484,647 |
| Leopard cat | 81 | 43 | - | - | 20,870,201 |

Cf. caption of Table S3.

Table S5: MCMCTree mean divergence time estimates

| **Node** | **Age mean (mya)** | **Age 95% HPD (mya)** | **Age range (mya)** | **Secondary calibration, soft-bounded (mya)** |
|---|---|---|---|---|
| ((Jungle cat, Puma), genus *Leopardus*) | 10.02 | 9.76-10.28 | 7.73-13.32 | 6.83-14.04 |
| (Jungle cat, Puma) | 8.46 | 7.48-9.43 | 5.59-10.89 | - |
| Base of *Leopardus* | 4.45 | 3.87-5.00 | 2.89-6.29 | - |
| ((Margay, Andean cat), Ocelot_USA) | 4.04 | 3.51-4.59 | 2.46-5.16 | - |
| (Margay, Andean cat) | 3.72 | 3.15-4.30 | 1.91-4.80 | - |
| (Pampas cat_Chile, subg. *Oncifelis*) | 3.83 | 3.29-4.40 | 2.32-5.09 | - |
| (Andean tiger cat 32451, ((Geoffroy's cat, Guigna), (Eastern tiger cat_Brazil, Southern tiger cat))) | 2.39 | 1.67-3.07 | 1.33-4.12 | - |
| ((Geoffroy's cat, Guigna), (Eastern tiger cat_Brazil, Southern tiger cat)) | 2.03 | 1.51-2.53 | 1.14-3.42 | - |
| (Geoffroy's cat, Guigna) | 1.62 | 1.15-2.09 | 0.72-2.78 | - |
| (Eastern tiger cat_Brazil, Southern tiger cat) | 1.46 | 0.94-1.96 | 0.27-2.75 | - |

Mean divergence time estimates from 16.186 local timetrees computed with MCMCTree, using a root age constraint taken from a Felidae timetree published by Li et al. (2016). We used a global clock for sequence evolution, the Jukes and Cantor (1969) model of nucleotide substitution and a prior substitution rate of $2.26 \times 10^{-9}$ substitutions per base pair per year (Wang et al. 2022). For each node, the 95% Highest Posterior Density (HPD) interval and minimum and maximum estimates are given.

Table S6: Fractions of introgression inferred with QuIBL

| Taxon 1 | Taxon 2 | Introgressed fraction (%) |
|---|---|---|
| Geoffroy's cat | Southern tiger cat | 39.64 |
| C. Am. tiger cat | Andean tiger cat 32454 | 26.32 |
| C. Am. tiger cat | Andean tiger cat 32451 | 26.10 |
| Ocelot (Zoo) | Ocelot (Brazil) | 23.16 |
| Ocelot (Brazil) | Ocelot (USA) | 22.98 |
| Guigna | Southern tiger cat | 17.22 |
| Pampas cat (Zoo) | Andean cat | 16.47 |
| Pampas cat (Chile) | Andean cat | 16.47 |
| Pampas cat (Zoo) | Margay | 16.47 |
| Pampas cat (Chile) | Margay | 16.47 |
| Pampas cat (Zoo) | Ocelot (Brazil) | 13.29 |
| Pampas cat (Zoo) | Ocelot (Zoo) | 13.29 |
| Pampas cat (Chile) | Ocelot (Brazil) | 13.29 |
| Pampas cat (Chile) | Ocelot (Zoo) | 13.29 |
| Pampas cat (Zoo) | Ocelot (USA) | 13.29 |
| Pampas cat (Chile) | Ocelot (USA) | 13.29 |
| Eastern tiger cat (Zoo) | Ocelot (USA) | 13.21 |
| Eastern tiger cat (Brazil) | Ocelot (USA) | 13.21 |
| Southern tiger cat | Ocelot (USA) | 13.21 |
| Eastern tiger cat (Zoo) | Ocelot (Brazil) | 13.21 |
| Eastern tiger cat (Brazil) | Ocelot (Brazil) | 13.21 |
| Southern tiger cat | Ocelot (Brazil) | 13.21 |
| Eastern tiger cat (Zoo) | Ocelot (Zoo) | 13.21 |
| Eastern tiger cat (Brazil) | Ocelot (Zoo) | 13.21 |
| Southern tiger cat | Ocelot (Zoo) | 13.21 |
| Geoffroy's cat | Ocelot (USA) | 13.21 |
| Guigna | Ocelot (USA) | 13.20 |
| C. Am. tiger cat | Ocelot (USA) | 13.20 |
| Ocelot (USA) | Andean tiger cat 32454 | 13.20 |
| Ocelot (USA) | Andean tiger cat 32451 | 13.20 |
| Geoffroy's cat | Ocelot (Brazil) | 13.20 |
| Geoffroy's cat | Ocelot (Zoo) | 13.20 |
| Guigna | Ocelot (Brazil) | 13.20 |
| Guigna | Ocelot (Zoo) | 13.20 |
| C. Am. tiger cat | Ocelot (Brazil) | 13.20 |
| Ocelot (Brazil) | Andean tiger cat 32454 | 13.20 |
| Ocelot (Brazil) | Andean tiger cat 32451 | 13.20 |
| C. Am. tiger cat | Ocelot (Zoo) | 13.20 |
| Ocelot (Zoo) | Andean tiger cat 32454 | 13.20 |
| Ocelot (Zoo) | Andean tiger cat 32451 | 13.20 |
| Eastern tiger cat (Brazil) | Geoffroy's cat | 11.39 |
| Eastern tiger cat (Zoo) | Geoffroy's cat | 11.37 |
| Andean cat | Ocelot (Brazil) | 9.26 |
| Andean cat | Ocelot (Zoo) | 9.26 |
| Andean cat | Ocelot (USA) | 9.26 |
| Ocelot (Zoo) | Andean cat | 9.26 |
| Ocelot (Brazil) | Andean cat | 9.26 |
| Ocelot (USA) | Andean cat | 9.26 |
| Eastern tiger cat (Zoo) | Andean cat | 8.98 |
| Eastern tiger cat (Brazil) | Andean cat | 8.98 |
| Geoffroy's cat | Andean cat | 8.98 |
| Guigna | Andean cat | 8.98 |
| Southern tiger cat | Andean cat | 8.98 |
| Andean cat | C. Am. tiger cat | 8.98 |
| Andean cat | Andean tiger cat 32454 | 8.98 |
| Andean cat | Andean tiger cat 32451 | 8.98 |
| Andean cat | Southern tiger cat | 8.98 |
| Andean cat | Eastern tiger cat (Brazil) | 8.98 |
| Andean cat | Eastern tiger cat (Zoo) | 8.98 |
| Andean cat | Guigna | 8.98 |
| Andean cat | Geoffroy's cat | 8.98 |
| C. Am. tiger cat | Andean cat | 8.98 |
| Andean tiger cat 32451 | Andean cat | 8.98 |
| Andean tiger cat 32454 | Andean cat | 8.98 |

| | | |
|---|---|---|
| Eastern tiger cat (Brazil) | Guigna | 8.35 |
| Guigna | Eastern tiger cat (Brazil) | 8.35 |
| Eastern tiger cat (Zoo) | Guigna | 8.28 |
| Guigna | Eastern tiger cat (Zoo) | 8.28 |
| Ocelot (Zoo) | Margay | 7.86 |
| Margay | Ocelot (Zoo) | 7.86 |
| Ocelot (USA) | Margay | 7.86 |
| Margay | Ocelot (USA) | 7.86 |
| Ocelot (Brazil) | Margay | 7.86 |
| Margay | Ocelot (Brazil) | 7.86 |
| C. Am. tiger cat | Margay | 7.74 |
| Andean tiger cat 32451 | Margay | 7.74 |
| Andean tiger cat 32454 | Margay | 7.74 |
| Margay | C. Am. tiger cat | 7.74 |
| Margay | Andean tiger cat 32454 | 7.74 |
| Margay | Andean tiger cat 32451 | 7.74 |
| Eastern tiger cat (Zoo) | Margay | 7.73 |
| Eastern tiger cat (Brazil) | Margay | 7.73 |
| Geoffroy's cat | Margay | 7.73 |
| Guigna | Margay | 7.73 |
| Southern tiger cat | Margay | 7.73 |
| Margay | Southern tiger cat | 7.73 |
| Margay | Eastern tiger cat (Brazil) | 7.73 |
| Margay | Eastern tiger cat (Zoo) | 7.73 |
| Margay | Guigna | 7.73 |
| Margay | Geoffroy's cat | 7.73 |
| Guigna | C. Am. tiger cat | 7.51 |
| C. Am. tiger cat | Guigna | 7.51 |
| Guigna | Andean tiger cat 32451 | 7.48 |
| Andean tiger cat 32451 | Guigna | 7.48 |
| Guigna | Andean tiger cat 32454 | 7.44 |
| Andean tiger cat 32454 | Guigna | 7.44 |
| Geoffroy's cat | C. Am. tiger cat | 6.09 |
| C. Am. tiger cat | Geoffroy's cat | 6.09 |
| Geoffroy's cat | Andean tiger cat 32454 | 5.79 |
| Andean tiger cat 32454 | Geoffroy's cat | 5.79 |
| Geoffroy's cat | Andean tiger cat 32451 | 5.68 |
| Andean tiger cat 32451 | Geoffroy's cat | 5.68 |
| Southern tiger cat | C. Am. tiger cat | 4.77 |
| C. Am. tiger cat | Southern tiger cat | 4.77 |
| Southern tiger cat | Andean tiger cat 32451 | 4.74 |
| Andean tiger cat 32451 | Southern tiger cat | 4.74 |
| Southern tiger cat | Andean tiger cat 32454 | 4.73 |
| Andean tiger cat 32454 | Southern tiger cat | 4.73 |
| Eastern tiger cat (Brazil) | Andean tiger cat 32454 | 4.34 |
| Andean tiger cat 32454 | Eastern tiger cat (Brazil) | 4.34 |
| Eastern tiger cat (Brazil) | Andean tiger cat 32451 | 4.33 |
| Andean tiger cat 32451 | Eastern tiger cat (Brazil) | 4.33 |
| Eastern tiger cat (Zoo) | Andean tiger cat 32454 | 4.29 |
| Andean tiger cat 32454 | Eastern tiger cat (Zoo) | 4.29 |
| Eastern tiger cat (Zoo) | Andean tiger cat 32451 | 4.29 |
| Andean tiger cat 32451 | Eastern tiger cat (Zoo) | 4.29 |
| Eastern tiger cat (Brazil) | C. Am. tiger cat | 4.28 |
| C. Am. tiger cat | Eastern tiger cat (Brazil) | 4.28 |
| Eastern tiger cat (Zoo) | C. Am. tiger cat | 4.27 |
| C. Am. tiger cat | Eastern tiger cat (Zoo) | 4.27 |

Fractions below 1% are not shown. The analysis was conducted with QuIBL (Edelman et al. 2019).

Table S7: Genome-wide pairwise nucleotide diversity/divergence ($\pi/D_{xy}$)

| | Margay | Southern tiger cat | Pampas cat (Zoo) | Andean cat | Ocelot (Brazil) | Ocelot (Zoo) | C. Am. tiger cat | Andean tiger cat 32451 | Andean tiger cat 32454 | Eastern tiger cat (Zoo) | Eastern tiger cat (Brazil) | Pampas cat (Chile) | Guigna | Geoffroy's cat | Puma | Ocelot (USA) |
|---|---|---|---|---|---|---|---|---|---|---|---|---|---|---|---|---|
| Margay | N/A | 0.78 | 0.77 | 0.75 | 0.72 | 0.74 | 0.78 | 0.78 | 0.78 | 0.77 | 0.78 | 0.83 | 0.79 | 0.74 | 1.75 | 0.76 |
| Southern tiger cat | 0.74 | N/A | 0.77 | 0.82 | 0.75 | 0.77 | 0.50 | 0.50 | 0.50 | 0.39 | 0.41 | 0.84 | 0.46 | 0.38 | 1.80 | 0.79 |
| Pampas cat (Zoo) | 0.74 | 0.74 | N/A | 0.79 | 0.75 | 0.77 | 0.77 | 0.77 | 0.77 | 0.75 | 0.76 | 0.40 | 0.78 | 0.73 | 1.74 | 0.78 |
| Andean cat | 0.72 | 0.77 | 0.76 | N/A | 0.75 | 0.78 | 0.82 | 0.82 | 0.82 | 0.80 | 0.82 | 0.87 | 0.83 | 0.76 | 1.79 | 0.80 |
| Ocelot (Brazil) | 0.69 | 0.71 | 0.72 | 0.72 | N/A | 0.46 | 0.75 | 0.75 | 0.75 | 0.73 | 0.75 | 0.81 | 0.76 | 0.71 | 1.74 | 0.47 |
| Ocelot (Zoo) | 0.71 | 0.73 | 0.74 | 0.73 | 0.43 | N/A | 0.77 | 0.77 | 0.77 | 0.75 | 0.77 | 0.84 | 0.78 | 0.73 | 1.77 | 0.47 |
| C. Am. tiger cat | 0.74 | 0.47 | 0.74 | 0.77 | 0.71 | 0.73 | N/A | 0.30 | 0.30 | 0.48 | 0.50 | 0.84 | 0.50 | 0.45 | 1.79 | 0.79 |
| Andean tiger cat 32451 | 0.74 | 0.46 | 0.74 | 0.77 | 0.71 | 0.72 | 0.28 | N/A | 0.29 | 0.48 | 0.50 | 0.84 | 0.50 | 0.45 | 1.79 | 0.79 |
| Andean tiger cat 32454 | 0.74 | 0.46 | 0.74 | 0.77 | 0.71 | 0.72 | 0.28 | 0.27 | N/A | 0.48 | 0.50 | 0.84 | 0.50 | 0.45 | 1.79 | 0.79 |
| Eastern tiger cat (Zoo) | 0.73 | 0.36 | 0.72 | 0.75 | 0.69 | 0.71 | 0.45 | 0.45 | 0.45 | N/A | 0.16 | 0.81 | 0.46 | 0.41 | 1.77 | 0.77 |
| Eastern tiger cat (Brazil) | 0.74 | 0.38 | 0.73 | 0.77 | 0.71 | 0.72 | 0.46 | 0.46 | 0.46 | 0.14 | N/A | 0.84 | 0.48 | 0.42 | 1.79 | 0.79 |
| Pampas cat (Chile) | 0.79 | 0.79 | 0.38 | 0.82 | 0.77 | 0.79 | 0.79 | 0.79 | 0.79 | 0.77 | 0.79 | N/A | 0.85 | 0.78 | 1.84 | 0.86 |
| Guigna | 0.75 | 0.43 | 0.74 | 0.78 | 0.72 | 0.73 | 0.47 | 0.47 | 0.47 | 0.43 | 0.44 | 0.80 | N/A | 0.41 | 1.80 | 0.80 |
| Geoffroy's cat | 0.71 | 0.36 | 0.71 | 0.73 | 0.68 | 0.69 | 0.43 | 0.43 | 0.43 | 0.39 | 0.40 | 0.75 | 0.39 | N/A | 1.71 | 0.74 |
| Puma | 1.69 | 1.73 | 1.69 | 1.73 | 1.69 | 1.71 | 1.73 | 1.73 | 1.73 | 1.71 | 1.73 | 1.78 | 1.74 | 1.67 | N/A | 1.79 |
| Ocelot (USA) | 0.72 | 0.74 | 0.75 | 0.75 | 0.45 | 0.44 | 0.74 | 0.74 | 0.74 | 0.72 | 0.74 | 0.81 | 0.75 | 0.70 | 1.73 | N/A |

The percentage of base differences are shown for all pairwise whole-genome comparisons. Data below the diagonal (yellow-green color scheme) are calculated from repeatmasked pseudohaploid consensus genomes. Data above the diagonal (yellow-blue color scheme) are calculated from unmasked pseudohaploid consensus genomes and represent, on average, a 5% increase to the repeatmasked data. The highest values are colored yellow while the lowest values are the most saturated green and blue. Canada lynx was used as reference genome.

Table S8: Mitochondrial pairwise nucleotide diversity/divergence ($\pi/D_{xy}$)

| | Eastern tiger cat (Zoo) | Pampas cat (Zoo) | Guigna | Southern tiger cat | Andean tiger cat 32454 | Andean tiger cat 32451 | Geoffroy's cat | Ocelot (Brazil) | Eastern tiger cat (Brazil) | Margay | Pampas cat (Chile) | Andean cat | Puma | Ocelot (USA) | Ocelot (Zoo) | C. Am. tiger cat |
|---|---|---|---|---|---|---|---|---|---|---|---|---|---|---|---|---|
| Eastern tiger cat (Zoo) | N/A | 0.06 | 0.18 | 0.16 | 0.18 | 0.21 | 0.20 | 0.20 | 0.04 | 0.14 | 0.09 | 0.19 | 0.27 | 0.19 | 0.20 | 0.18 |
| Pampas cat (Zoo) | 0.74 | N/A | 0.19 | 0.18 | 0.20 | 0.21 | 0.22 | 0.23 | 0.06 | 0.18 | 0.09 | 0.19 | 0.27 | 0.21 | 0.22 | 0.20 |
| Guigna | 7.69 | 7.74 | N/A | 0.16 | 0.16 | 0.14 | 0.12 | 0.18 | 0.20 | 0.18 | 0.17 | 0.16 | 0.28 | 0.20 | 0.20 | 0.17 |
| Southern tiger cat | 7.44 | 7.28 | 5.31 | N/A | 0.12 | 0.13 | 0.16 | 0.17 | 0.18 | 0.15 | 0.18 | 0.19 | 0.27 | 0.17 | 0.20 | 0.14 |
| Andean tiger cat 32454 | 7.46 | 7.44 | 5.36 | 4.20 | N/A | 0.03 | 0.15 | 0.14 | 0.20 | 0.16 | 0.19 | 0.13 | 0.27 | 0.14 | 0.17 | 0.10 |
| Andean tiger cat 32451 | 7.34 | 7.40 | 5.20 | 4.15 | 0.28 | N/A | 0.17 | 0.15 | 0.22 | 0.16 | 0.20 | 0.14 | 0.26 | 0.13 | 0.15 | 0.09 |
| Geoffroy's cat | 7.53 | 7.56 | 3.20 | 5.33 | 5.27 | 5.08 | N/A | 0.18 | 0.21 | 0.21 | 0.20 | 0.17 | 0.28 | 0.23 | 0.24 | 0.17 |
| Ocelot (Brazil) | 7.01 | 7.01 | 7.12 | 6.79 | 6.79 | 6.77 | 6.97 | N/A | 0.21 | 0.14 | 0.18 | 0.13 | 0.26 | 0.10 | 0.08 | 0.16 |
| Eastern tiger cat (Brazil) | 0.39 | 0.58 | 7.66 | 7.35 | 7.38 | 7.32 | 7.49 | 6.93 | N/A | 0.17 | 0.10 | 0.20 | 0.26 | 0.20 | 0.21 | 0.20 |
| Margay | 6.91 | 6.86 | 7.12 | 6.64 | 6.75 | 6.75 | 6.94 | 4.20 | 6.75 | N/A | 0.18 | 0.11 | 0.24 | 0.20 | 0.21 | 0.18 |
| Pampas cat (Chile) | 2.26 | 2.11 | 7.71 | 7.44 | 7.39 | 7.38 | 7.61 | 7.01 | 2.09 | 6.65 | N/A | 0.21 | 0.24 | 0.17 | 0.18 | 0.19 |
| Andean cat | 6.89 | 6.99 | 7.30 | 7.02 | 7.19 | 7.13 | 7.13 | 6.69 | 6.86 | 6.63 | 7.00 | N/A | 0.24 | 0.14 | 0.14 | 0.17 |
| Puma | 10.04 | 9.94 | 10.16 | 10.04 | 9.97 | 9.99 | 10.05 | 9.49 | 9.90 | 9.70 | 10.01 | 9.67 | N/A | 0.27 | 0.25 | 0.27 |
| Ocelot (USA) | 6.90 | 6.94 | 6.80 | 6.84 | 6.78 | 6.61 | 6.71 | 2.12 | 6.86 | 4.19 | 6.93 | 6.68 | 9.61 | N/A | 0.06 | 0.15 |
| Ocelot (Zoo) | 6.89 | 6.91 | 6.71 | 7.08 | 6.91 | 6.76 | 6.76 | 2.19 | 6.87 | 4.31 | 6.91 | 6.69 | 9.55 | 1.10 | N/A | 0.17 |
| C. Am. tiger cat | 7.54 | 7.51 | 5.42 | 4.38 | 1.34 | 1.35 | 5.42 | 6.91 | 7.47 | 6.71 | 7.51 | 7.31 | 10.04 | 6.92 | 7.01 | N/A |

The number of base differences per base pair are shown for pairwise mitogenome comparisons (yellow-green color scheme). The highest values are colored yellow while the lowest values are the most saturated green. Standard error estimates from 500 bootstrap replicates are shown above the diagonal. All positions with less than 50% site coverage were eliminated. That is, fewer than 50% alignment gaps were allowed at any position. There were a total of 17.328 positions in the final dataset. The analysis was conducted in MEGA7 (Kumar et al. 2016).

Table S9: Autosomal heterozygosity and ROHs (Canada lynx reference)

| Sample | ROHan mean heterozygosity (%) incl. ROHs | ROHan mean heterozygosity (%) excl. ROHs | ROH (%) | mean ROH length (Mb) | ANGSD mean heterozygosity (%) | ANGSD IQR (%) | VariantQC mean heterozygosity (%) |
|---|---|---|---|---|---|---|---|
| Ocelot (USA) | 0.421 | 0.421 | 0.0 | N/A | 0.289 | 0.086 | 0.416 |
| Ocelot (Brazil) | 0.409 | 0.409 | 0.0 | N/A | 0.272 | 0.087 | 0.392 |
| Ocelot (Zoo) | 0.404 | 0.406 | 0.8 | 2.7 | 0.256 | 0.087 | 0.384 |
| Leopard cat | 0.314 | 0.314 | 0.0 | N/A | 0.192 | 0.082 | 0.314 |
| Southern tiger cat | 0.279 | 0.280 | 0.3 | 0.2 | 0.156 | 0.077 | 0.242 |
| Geoffroy's cat | 0.248 | 0.248 | 0.3 | N/A | 0.151 | 0.057 | 0.214 |
| Andean tiger cat 32454 | 0.235 | 0.235 | 0.2 | 2.7 | 0.127 | 0.067 | 0.198 |
| Andean tiger cat 32451 | 0.233 | 0.236 | 1.8 | 4.4 | 0.127 | 0.066 | 0.200 |
| Margay | 0.232 | 0.250 | 6.7 | 9.5 | 0.141 | 0.100 | 0.203 |
| Pampas cat (Zoo) | 0.188 | 0.223 | 18.6 | 15.1 | 0.111 | 0.165 | 0.162 |
| C. Am. tiger cat | 0.144 | 0.144 | 0.0 | N/A | 0.076 | 0.099 | 0.108 |
| Eastern tiger cat (Zoo) | 0.124 | 0.124 | 0.0 | N/A | 0.061 | 0.043 | 0.086 |
| Pampas cat (Chile) | 0.107 | 0.107 | 0.0 | N/A | 0.045 | 0.030 | 0.071 |
| Eastern tiger cat (Brazil) | 0.101 | 0.102 | 0.7 | 15.0 | 0.046 | 0.050 | 0.066 |
| Guigna | 0.094 | 0.094 | 0.0 | N/A | 0.036 | 0.036 | 0.056 |
| Andean cat | 0.066 | 0.066 | 0.0 | N/A | 0.020 | 0.014 | 0.031 |
| Iberian lynx | 0.048 | 0.055 | 15.7 | 9.1 | 0.027 | 0.029 | 0.035 |

Average autosomal heterozygosity is shown in percentage of heterozygous sites, as estimated for individual, unphased samples with ROHan (Renaud 2019) and ANGSD (Korneliussen et al. 2014). For estimates with ROHan, heterozygosity is shown excluding and including ROHs, as well as the percentage of the genome in ROHs and the mean ROH length. For local (200kb) estimates of heterozygosity with ANGSD, the interquartile range (IQR) of all local values is shown. Heterozygosity from the SNP data set was calculated with VariantQC (Yan et al. 2019). Canada lynx was used as reference genome.

Table S10: Autosomal heterozygosity and ROHs (Geoffroy's cat reference)

| Sample | ROHan mean heterozygosity (%) incl. ROHs | ROHan mean heterozygosity (%) excl. ROHs | ROH (%) | mean ROH length (Mb) | ANGSD mean heterozygosity (%) | ANGSD IQR (%) | VariantQC mean heterozygosity (%) |
|---|---|---|---|---|---|---|---|
| Ocelot (USA) | 0.408 | 0.408 | 0.0 | N/A | 0.390 | 0.148 | 0.414 |
| Ocelot (Brazil) | 0.396 | 0.397 | 0.1 | 1.0 | 0.356 | 0.136 | 0.395 |
| Ocelot (Zoo) | 0.391 | 0.395 | 1.0 | 2.9 | 0.344 | 0.138 | 0.382 |
| Leopard cat | 0.313 | 0.313 | 0.0 | N/A | 0.182 | 0.066 | 0.312 |
| Southern tiger cat | 0.253 | 0.258 | 1.2 | 2.3 | 0.228 | 0.130 | 0.239 |
| Geoffroy's cat | 0.217 | 0.218 | 0.3 | 8.0 | 0.199 | 0.095 | 0.214 |
| Margay | 0.217 | 0.240 | 9.1 | 8.4 | 0.181 | 0.140 | 0.199 |
| Andean tiger cat 32451 | 0.210 | 0.217 | 2.6 | 4.0 | 0.188 | 0.114 | 0.191 |
| Andean tiger cat 32454 | 0.209 | 0.213 | 2.2 | 2.2 | 0.187 | 0.114 | 0.189 |
| Pampas cat (Zoo) | 0.168 | 0.232 | 29.0 | 9.7 | 0.143 | 0.222 | 0.153 |
| C. Am. tiger cat | 0.118 | 0.131 | 10.0 | 7.0 | 0.103 | 0.144 | 0.096 |
| Eastern tiger cat (Zoo) | 0.099 | 0.099 | 0.0 | N/A | 0.079 | 0.064 | 0.073 |
| Pampas cat (Chile) | 0.088 | 0.094 | 3.2 | 5.0 | 0.061 | 0.043 | 0.058 |
| Eastern tiger cat (Brazil) | 0.074 | 0.092 | 23.8 | 17.8 | 0.061 | 0.076 | 0.052 |
| Iberian lynx | 0.073 | 0.073 | 0.0 | N/A | 0.025 | 0.021 | 0.046 |
| Guigna | 0.063 | 0.070 | 10.7 | 5.1 | 0.049 | 0.055 | 0.041 |
| Andean cat | 0.044 | 0.044 | 0.8 | 15.0 | 0.026 | 0.020 | 0.018 |

Cf. caption of Table S8. Geoffroy's cat was used as a reference genome.

3 SUPPLEMENTARY FIGURES

Figure S1: Summary trees inferred from unmasked genomes mapped to Canada lynx reference

A) Greedy consensus of 23,007 local NJ trees, with node frequency support. B) Greedy consensus of 23.007 local ML trees, with node frequency support. C) ASTRAL consensus of the NJ tree set, with local PP support. D) ASTRAL consensus of the ML tree set, with local PP support. E) Bootstrapped NJ tree from the genome-wide distance matrix, which is the sum total of all local distance matrices. F) Topology obtained in A-C and E. G) Topology obtained in D.

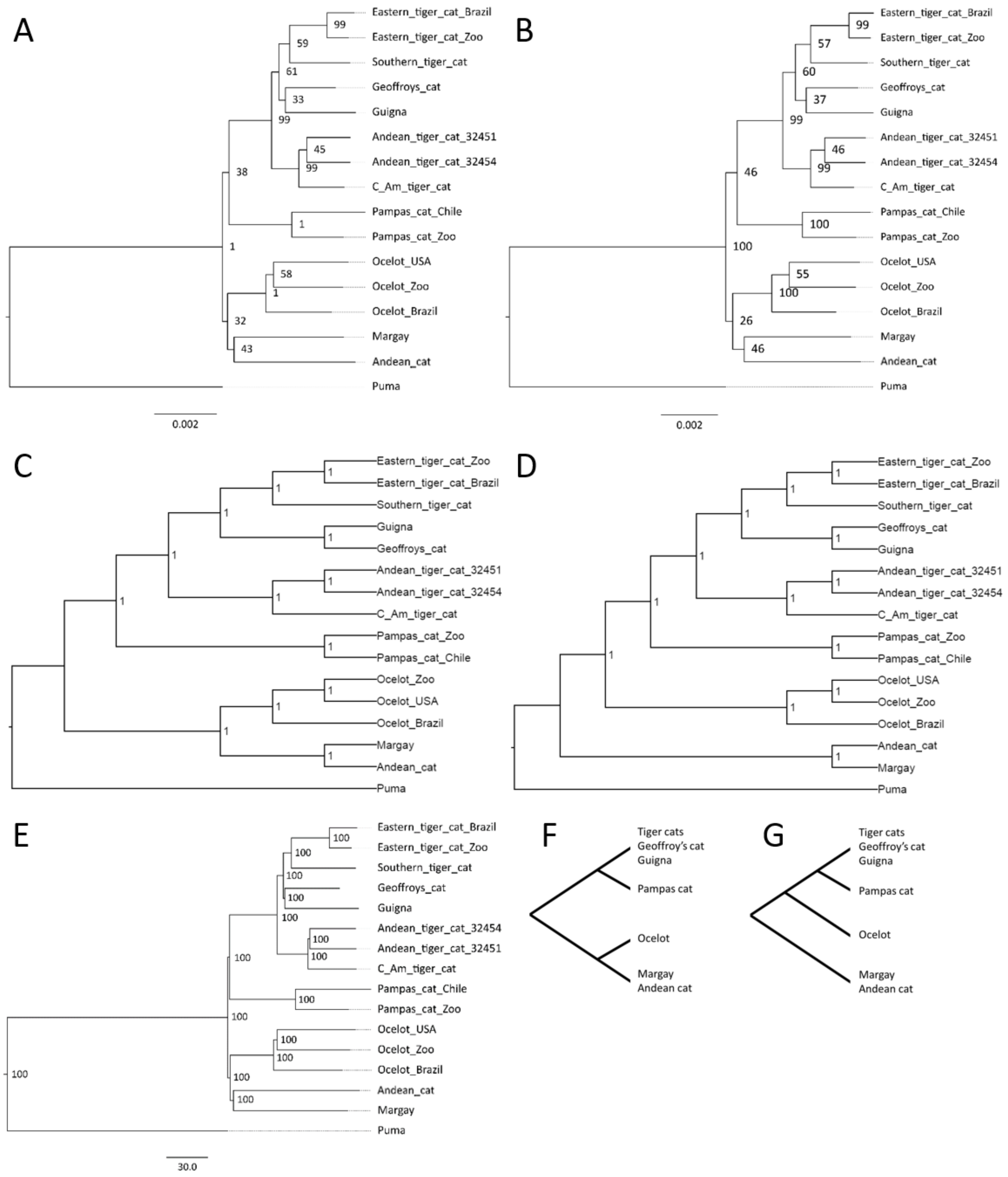

Figure S2: Summary trees inferred from repeatmasked genomes mapped to Geoffroy's cat reference

A) Greedy consensus of 21,028 local NJ trees, with node frequency support. B) Greedy consensus of 21,028 local ML trees, with node frequency support. C) ASTRAL consensus of the NJ tree set, with local PP support. D) ASTRAL consensus of the ML tree set, with local PP support. E) Bootstrapped NJ tree from the genome-wide distance matrix, which is the sum total of all local distance matrices. F) Overall topology obtained in A-E. G) Geoffroy's cat position in A-D. H) Geoffroy's cat position in E.

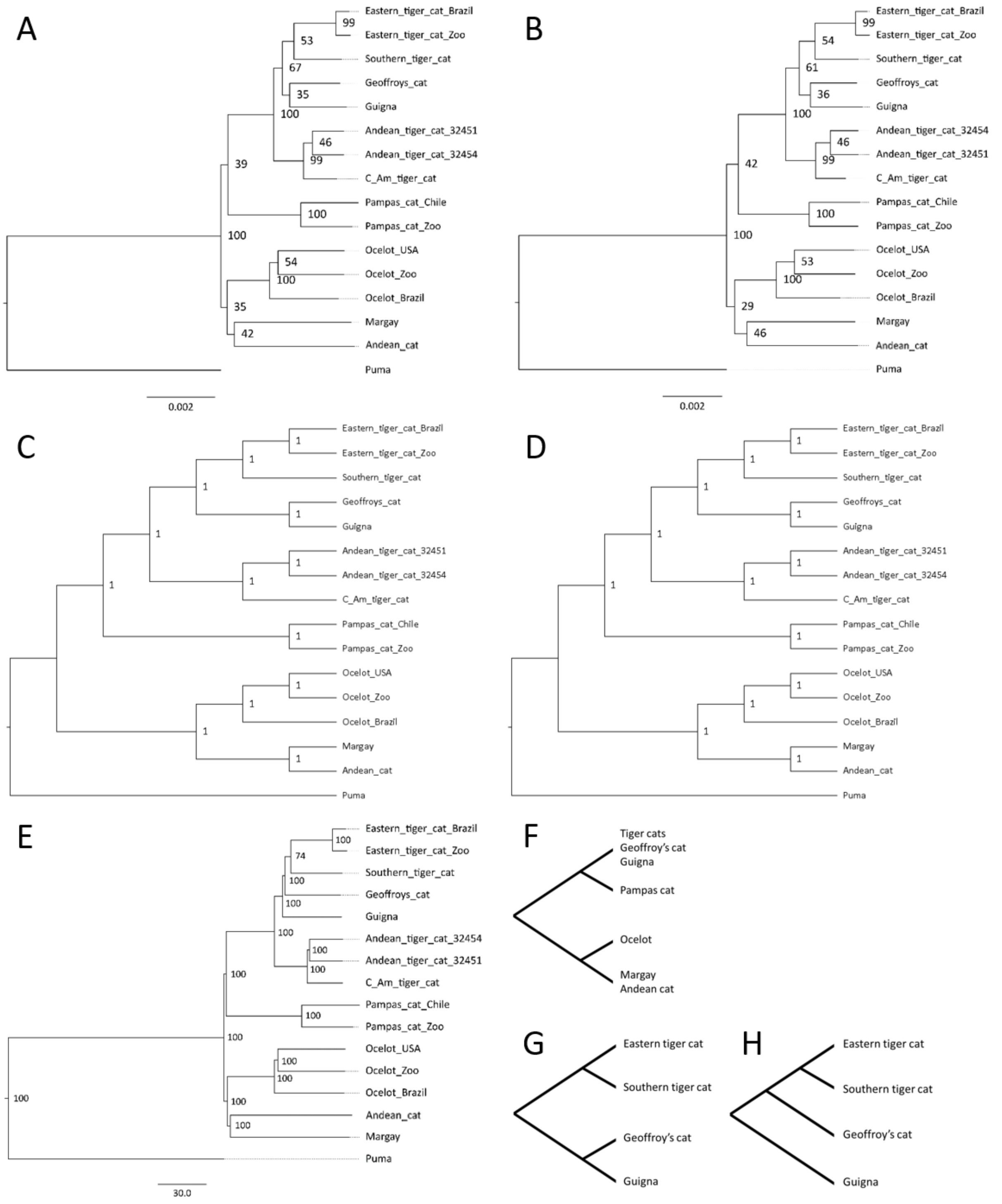

Figure S3: Summary trees inferred from unmasked genomes mapped to Geoffroy's cat reference

A) Greedy consensus of 23,136 local NJ trees, with node frequency support. B) Greedy consensus of 23,136 local ML trees, with node frequency support. C) ASTRAL consensus of the NJ tree set, with local PP support. D) ASTRAL consensus of the ML tree set, with local PP support. E) NJ tree from the genome-wide distance matrix, which is the sum total of all local distance matrices. F) Topology obtained in A-E.

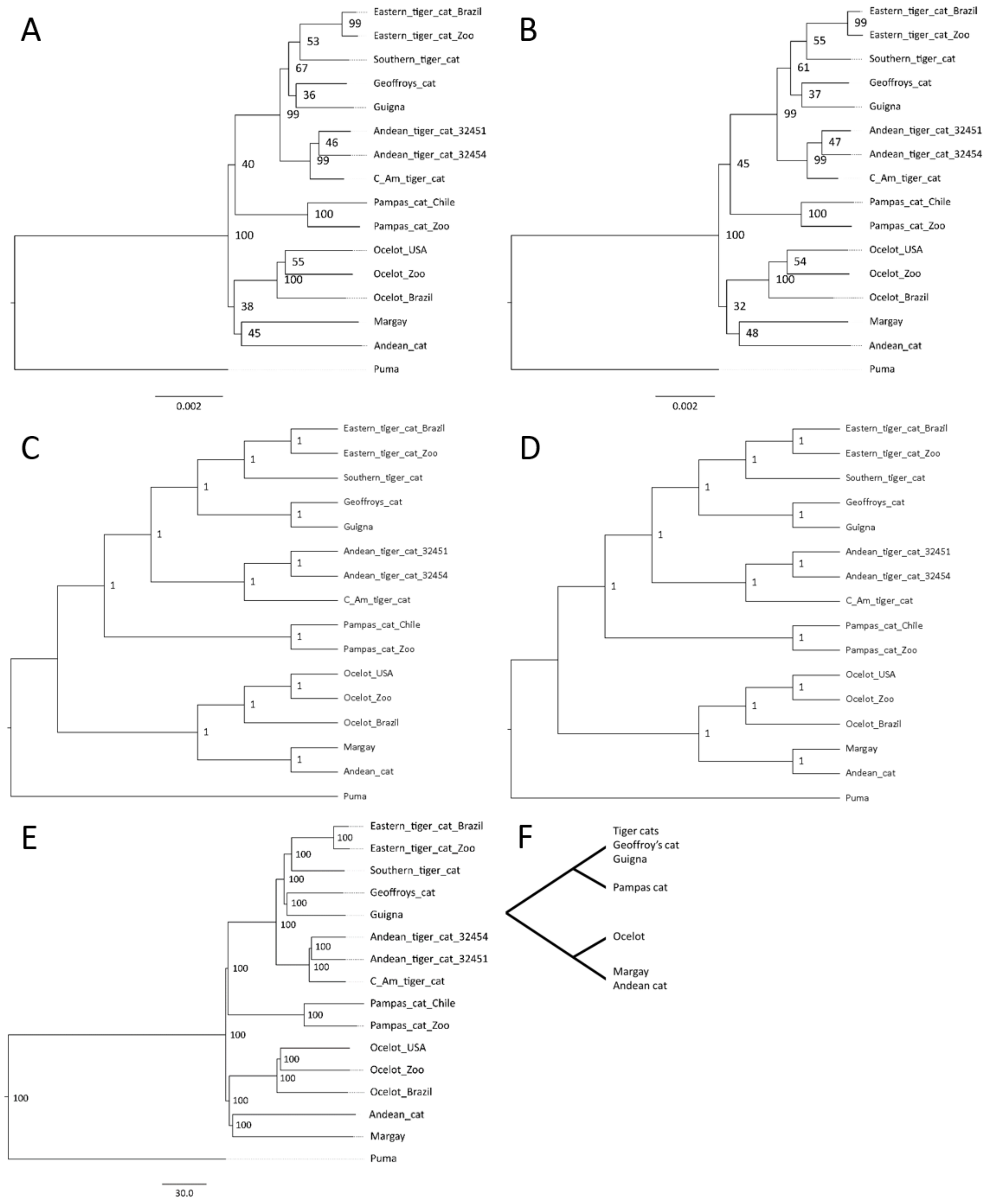

Figure S4: Estimates of the time of divergence between Andean tiger cat and C. Am. tiger cat

A) Demographic history derived from samples 'Andean tiger cat 32451' and 'C. Am. tiger cat' with MSMC2, shown as effective population sizes over the last 2 million years. Samples were mapped to the Geoffroy's cat reference. B) Relative cross-coalescent rate (rCCR) between both samples, computed with MSMC2. Conventionally, 50% of the maximum rCCR is taken to be the time of divergence between the two populations, which here corresponds to 157 kya. C) Isolation-Migration model applied to coalescence rates with MSMC-IM, showing the inferred cumulative distribution function of migration, M(t). Dotted blue lines represent 25%, 50% and 75% probability that populations are completely admixed. The 50% probability value is the best estimate of divergence time, with the 25-75% interval used to express uncertainty. The divergence time is estimated at 171 (61-453) kya.

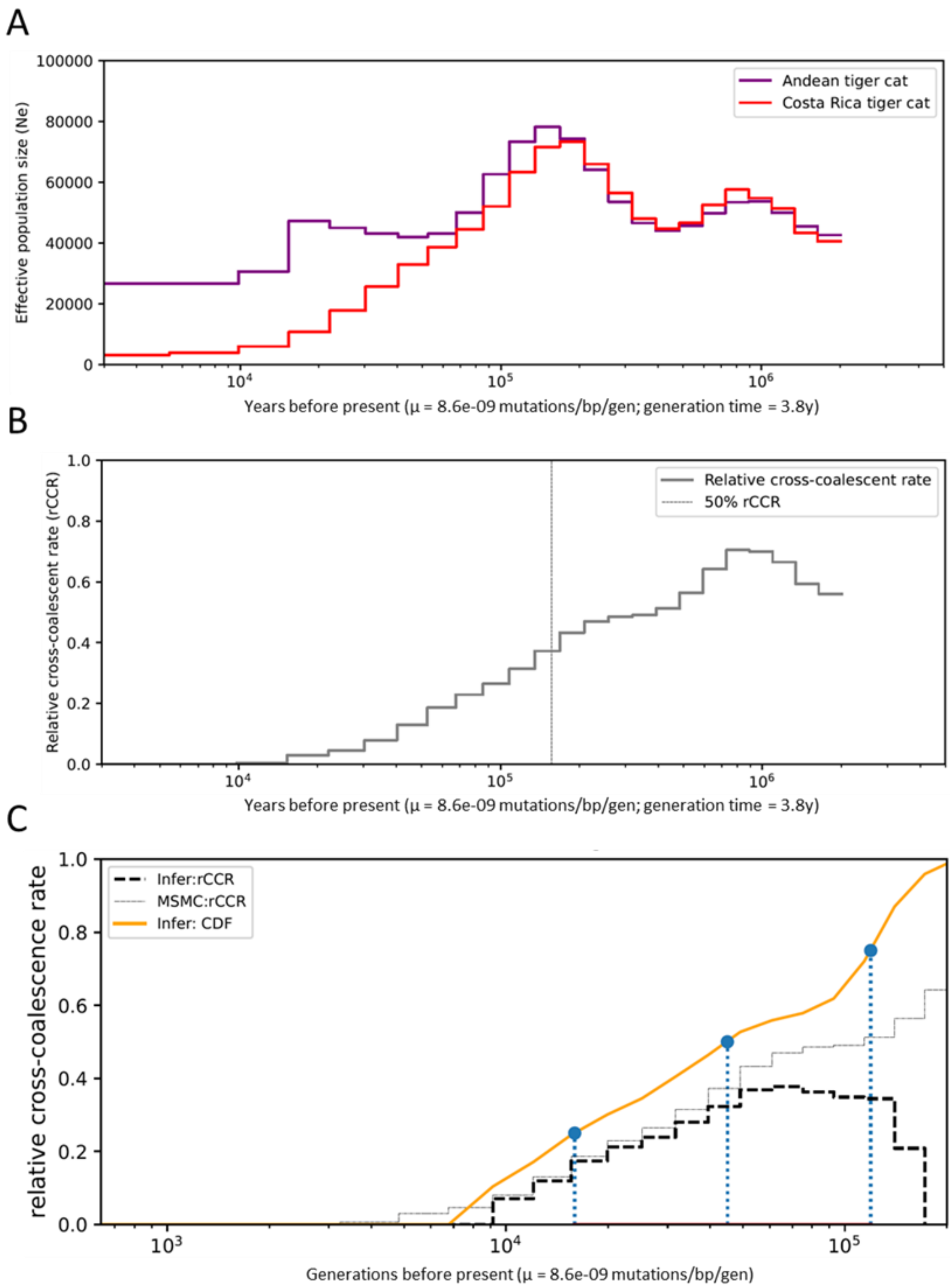

Figure S5: Fitting of the Isolation-Migration model with MSMC-IM

The MSMC-IM model was run on coalescence rates for samples ‘Andean tiger cat 32451’ and ‘C. Am. tiger cat’ as obtained with MSMC2. We explored 16 different values for the fitting parameter “-beta”, centered around the default value of b1=1×$10^{-8}$, b2=1×$10^{-6}$ (default result indicated in red and shown in Figure S4C). For each “-beta” setting, the yellow line shows the cumulative distribution function of migration, M(t). The thin, solid line shows the MSMC rCCR and the dashed line the MSMC-IM rCCR. When converted to years, the full range of time points at 50% of the M(t), subject to different values of “-beta”, amounts to 81-176 kya. For 25%, this is 44-61 kya, for 75% this is 89-470 kya.

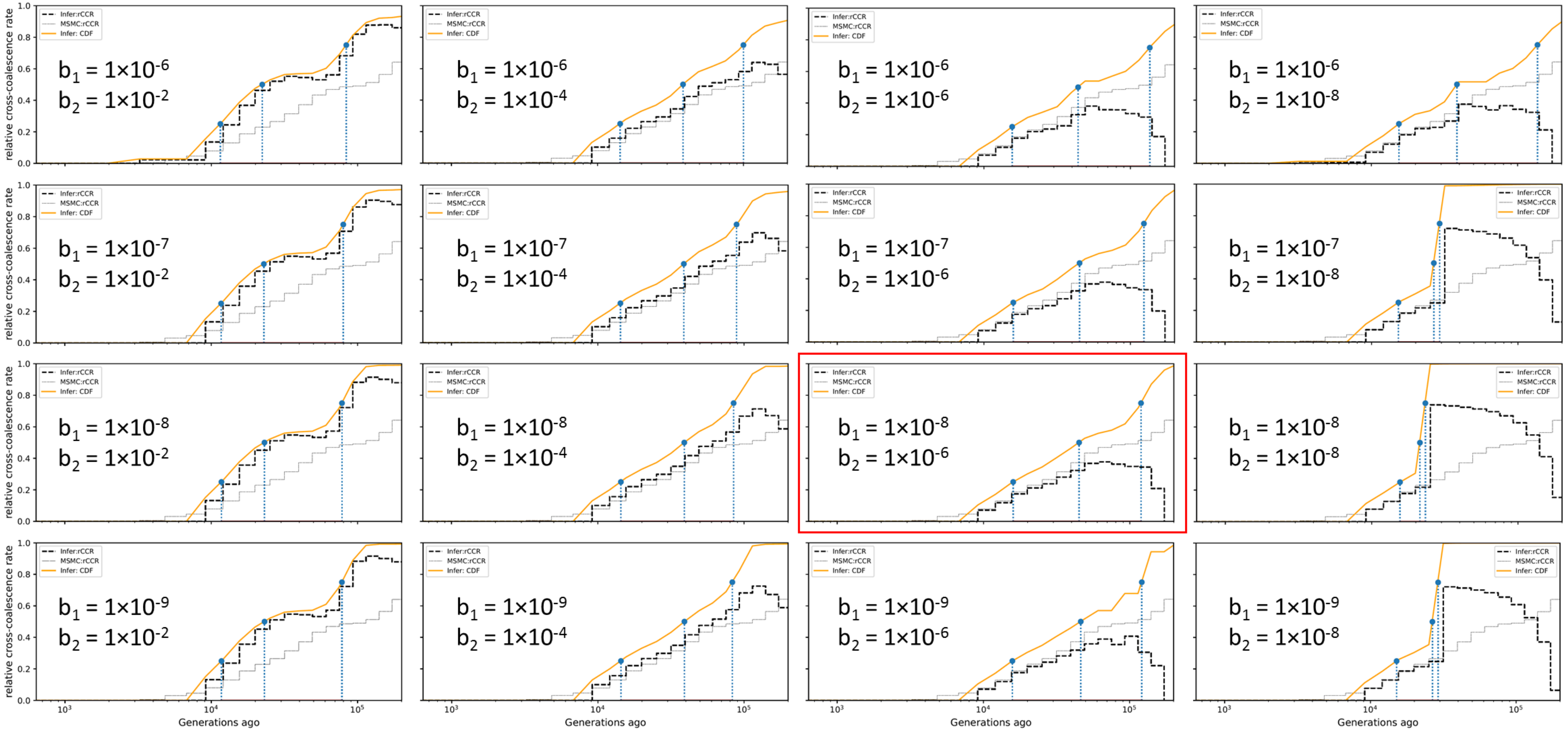

Figure S6: Branch Attachment Frequencies in the ML tree set

Branch Attachment Frequencies (BAF) for a selection of samples (one representative for each species-level population) in a set of 16.186 local ML trees. The BAF indicates the frequency with which a given sample (color-coded) is found attached to a given branch, in the set of local trees. BAFs <1% are not shown. Samples were mapped to the Canada lynx reference and repeatmasked.

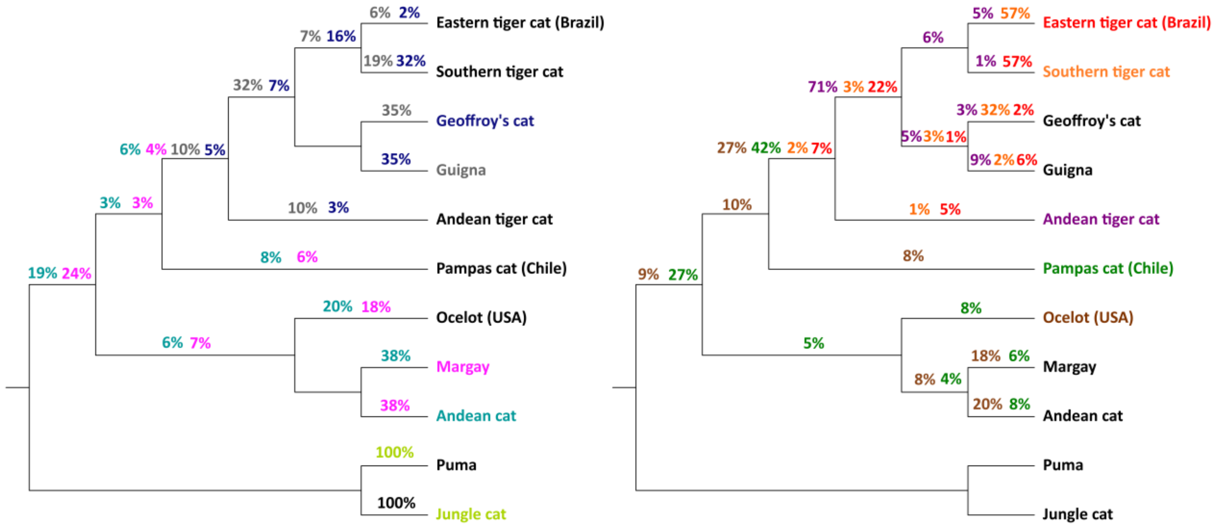


Figure S7: Implicit consensus network of the NJ tree set

Implicit network generated from a random subset of 3,268 local NJ trees (20% of the full set) with the Consensus Network algorithm in SplitsTree5 (Huson and Bryant 2006) using median edge weights and a minimum threshold of 20% for inclusion of edges. Samples were mapped to the Canada lynx reference and repeatmasked.

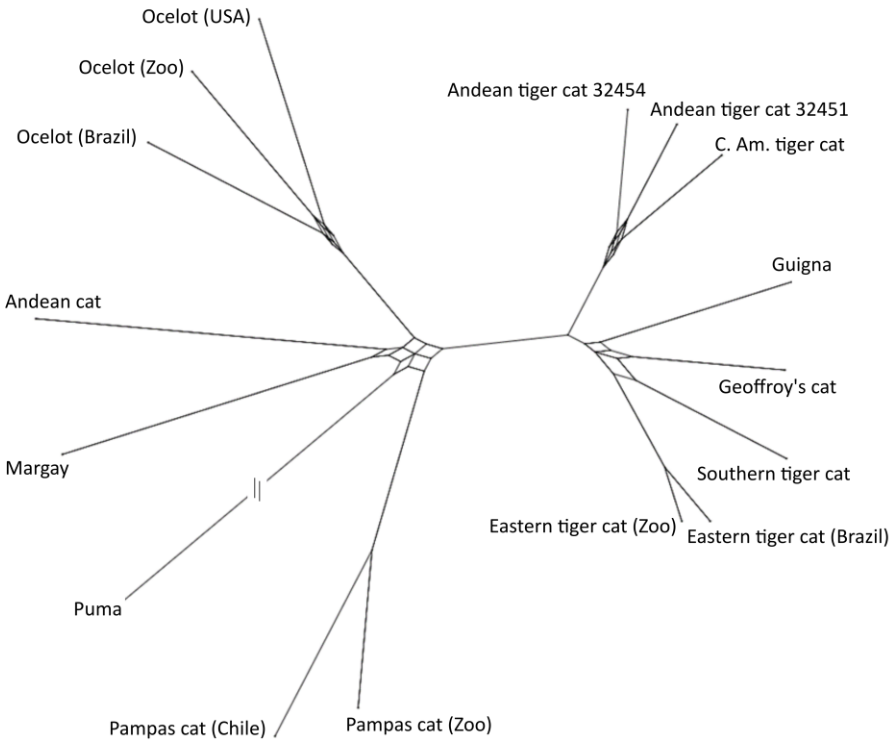

Figure S8: Pairwise average $f_4$-ratios, with the Canada lynx reference

Genome-wide pairwise *f4*-ratios, computed from the SNP data set. Colors correspond to statistical support (light to dark) and magnitude (blue to red) of the *f4*-ratio. Samples were mapped to the Canada lynx reference and repeatmasked.

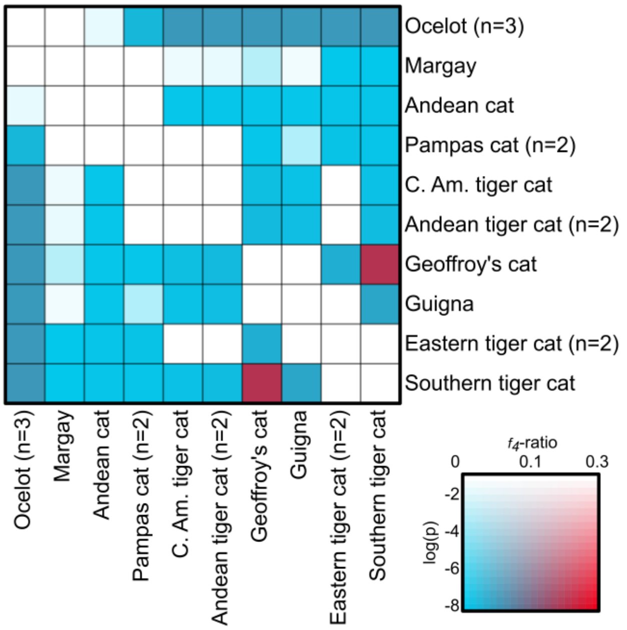


Figure S9: Pairwise average Patterson's D, with the Geoffroy's cat reference

Genome-wide pairwise Patterson's D, calculated from ABBA/BABA site patterns in the SNP data set. Colors correspond to statistical support (light to dark) and magnitude (blue to red) of the D-statistic. Samples were mapped to the Geoffroy's cat reference and repeatmasked.

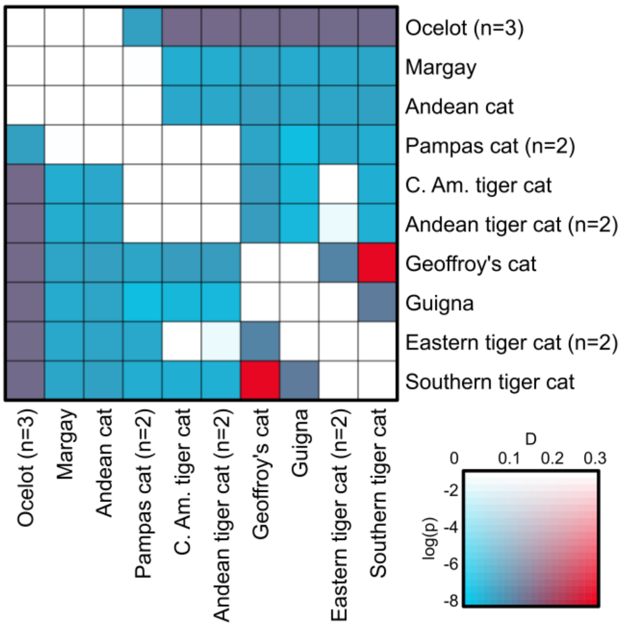

Figure S10: Pairwise average $f_4$-ratios, with the Geoffroy's cat reference

Genome-wide pairwise *f4*-ratios, computed from the SNP data set. Colors correspond to statistical support (light to dark) and magnitude (blue to red) of the *f4*-ratio. Samples were mapped to the Geoffroy's cat reference and repeatmasked.

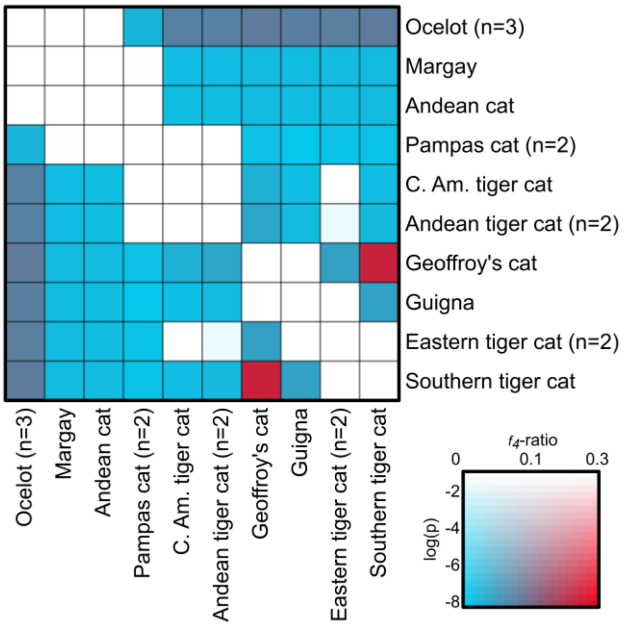


Figure S11: Pairwise *f*-branch-statistics, with the Geoffroy's cat reference

Pairwise *f*-branch (*fb*) statistic as a measure of the fraction of introgression between extant and ancestral populations or species, inferred from the SNP data set. Samples were mapped to the Geoffroy's cat reference and repeatmasked.

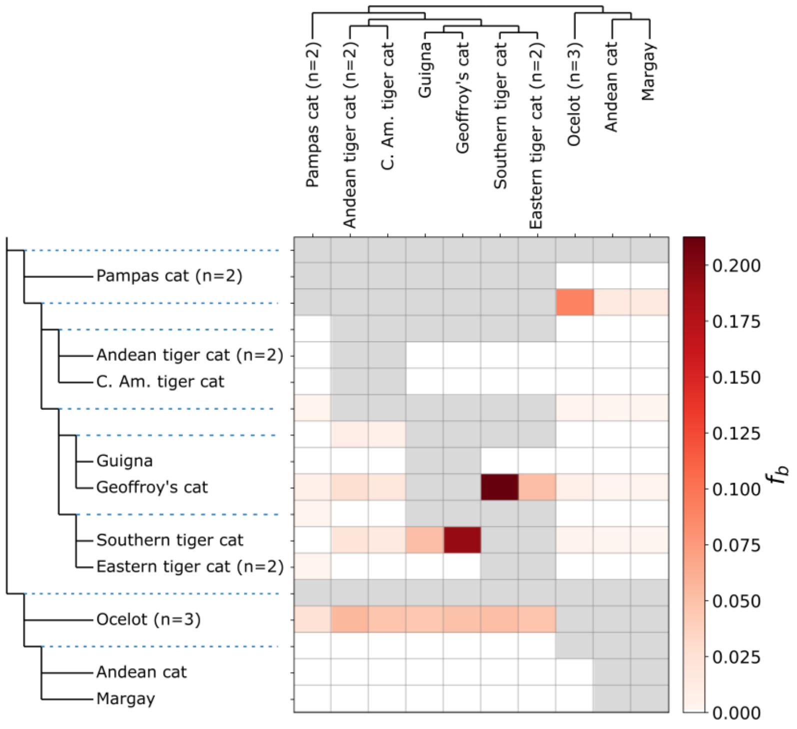

Figure S12: Slope heuristic and phylogenetic networks, with the Canada lynx reference

A) Slope of the negative log-pseudolikelihood of the optimal network under different numbers of hybrid edges (0-3). B-E) Optimal phylogenetic network after 50 iterations, constrained to 0, 1, 2 or 3 hybrid edges. Samples were mapped to the Canada lynx reference and repeatmasked.

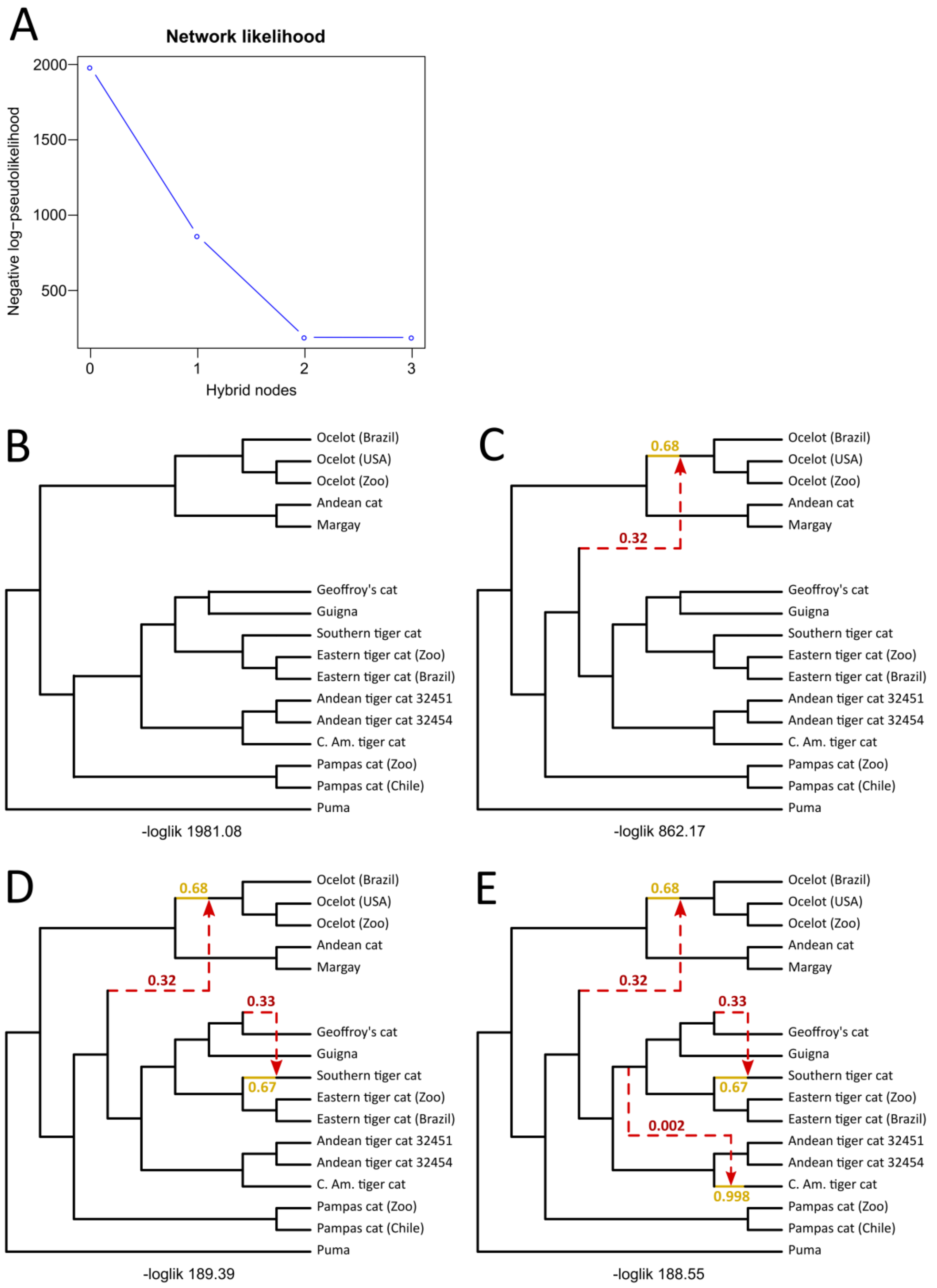

Figure S13: Slope heuristic and selected phylogenetic network, with the Geoffroy's cat reference

Optimal phylogenetic network inferred with PhyloNetworks (Solís-Lemus et al. 2017) under a maximum of 2 hybrid edges, using a single representative sample per (sub)species and the ML tree set as input.

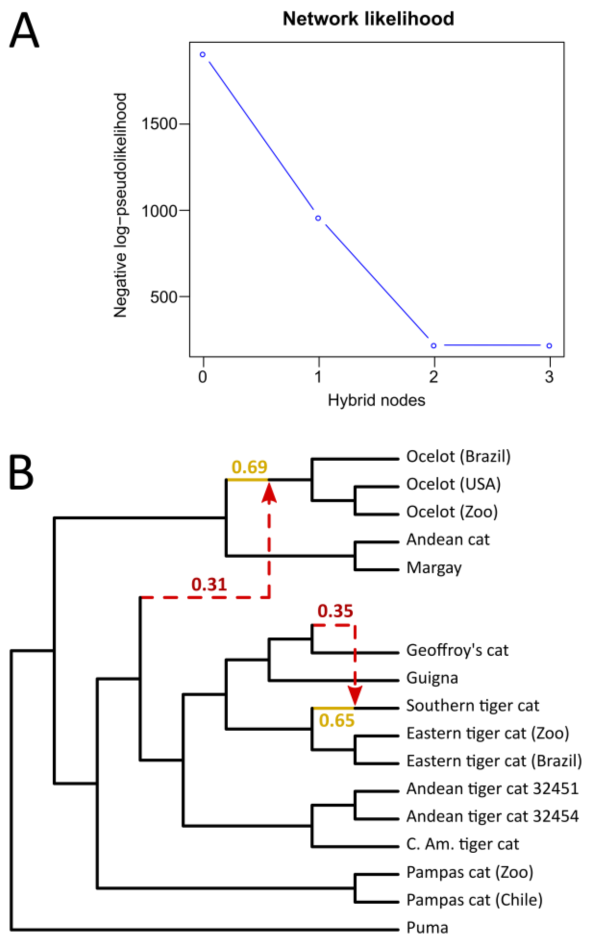

Figure S14: Principal Component Analysis (PCA), with the Canada lynx reference

(A) PCA with all *Leopardus* samples; (B) PCA with a subset comprising samples from the subgenera *Leopardus* and *Lynchailurus*; and (C) PCA with a subset comprising samples from the subgenus *Oncifelis*. The Canada lynx was used as a reference genome.

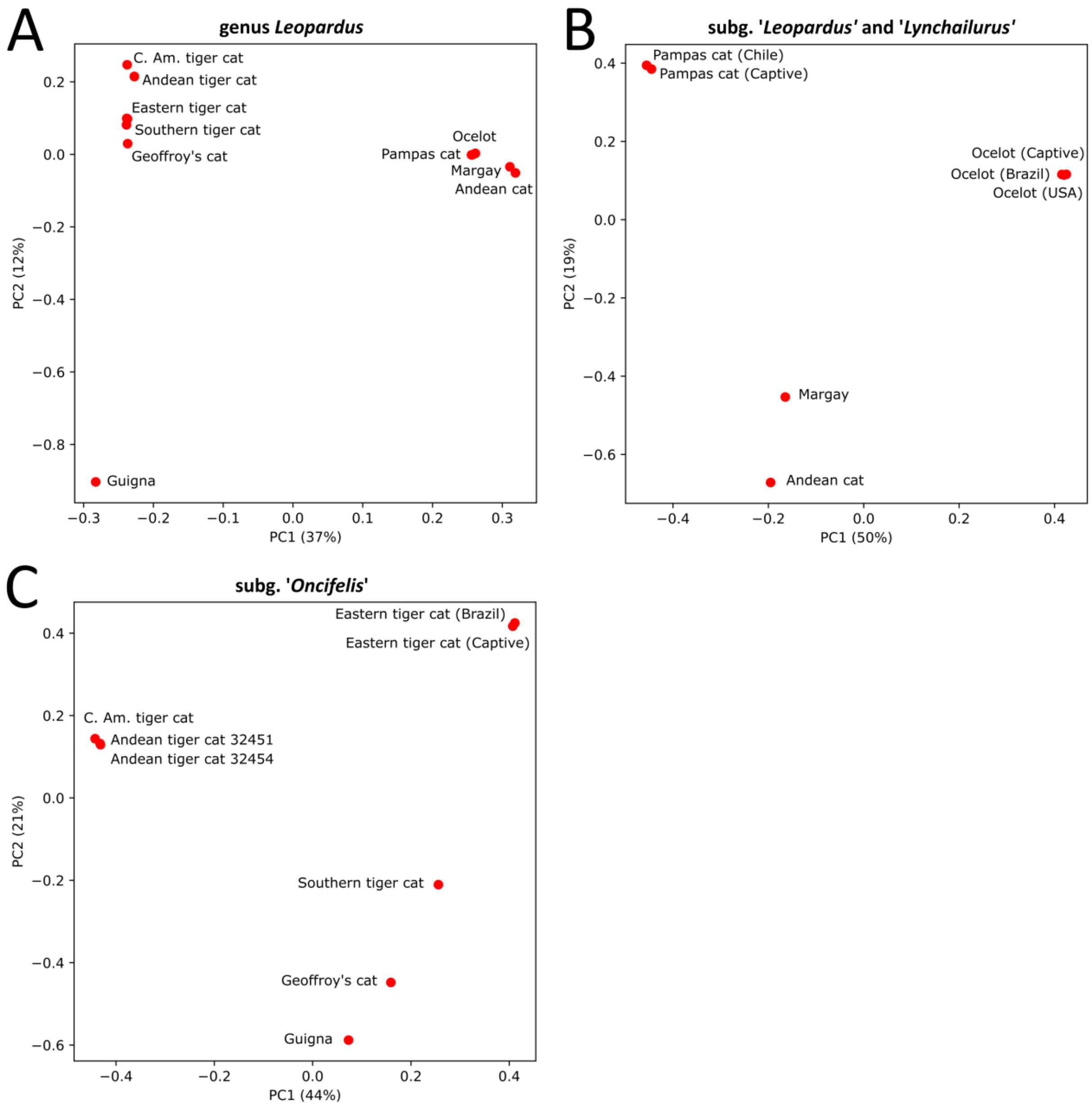

Figure S15: Runs of homozygosity in all autosomes of sample ‘C. Am. tiger cat’.

Bayesian inference of ROHs along all autosomes in sample ‘C. Am. tiger cat’. Regions with a low posterior probability (low on y-axis) are identified as ROHs, equivalent to 10.0% of the autosomal part of the genome in this sample. The green line in the ROH plots denotes inference from the point estimates of local heterozygosity rates, the magenta and red lines denote inference from the upper resp. lower bounds of the heterozygosity estimates. Reads were mapped to the congeneric Geoffroy’s cat reference.

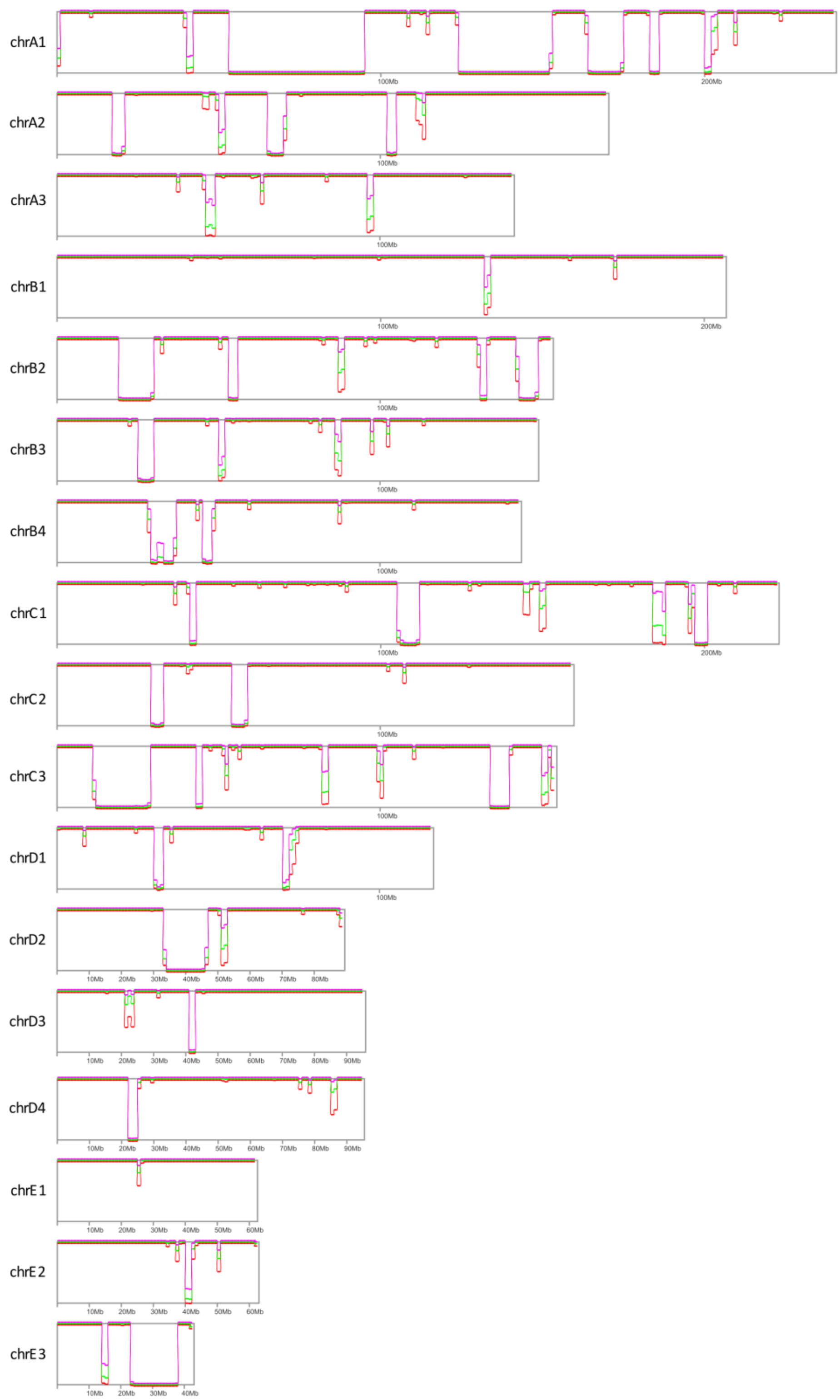

Figure S16: Runs of homozygosity in all autosomes of sample ‘Andean cat’.

Bayesian inference of ROHs along all autosomes in sample ‘Andean cat’, equivalent to 0.8% of the autosomal part of the genome in this sample. Caption cfr. Figure S15.

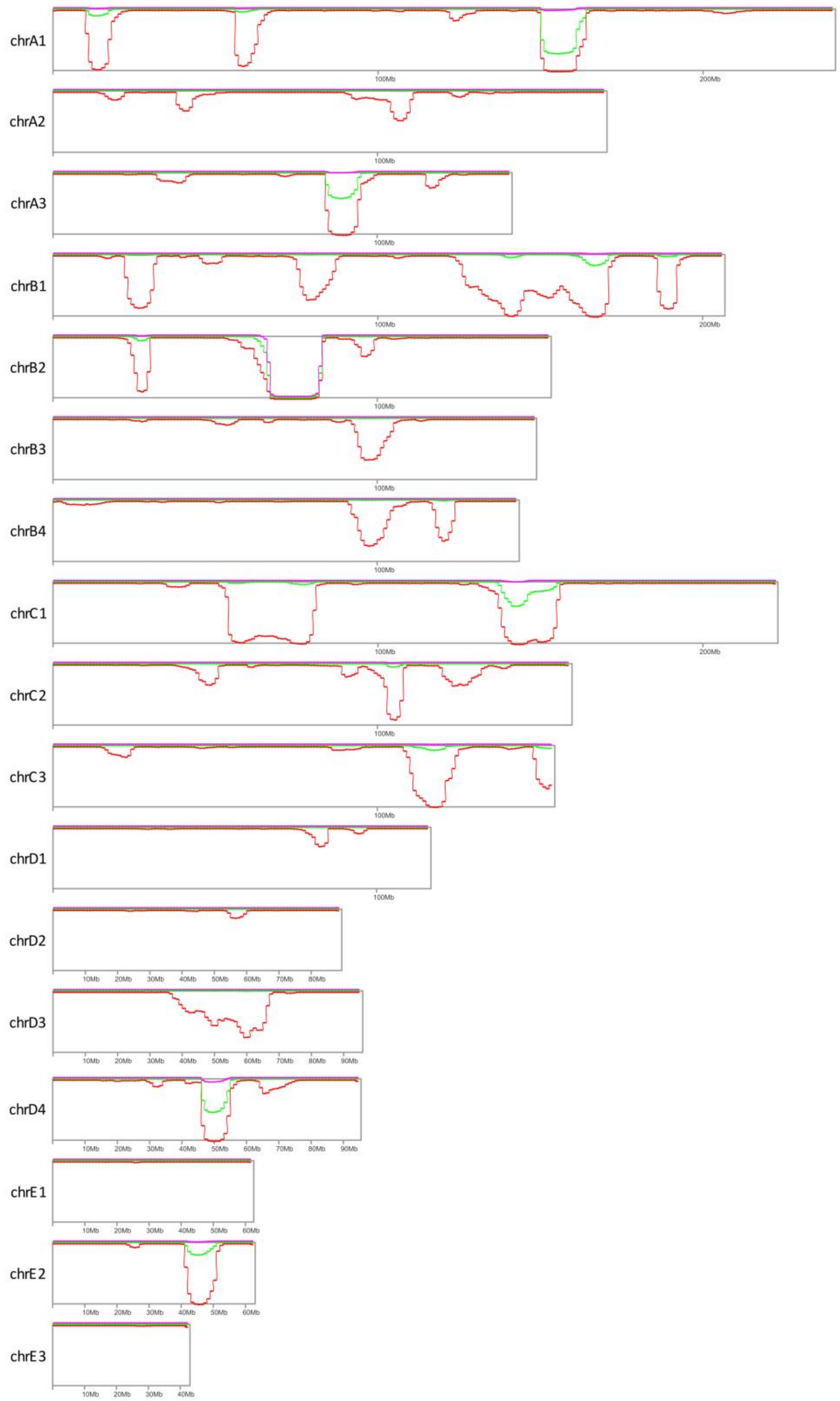

Figure S17: Demographic history with bootstrap replicates.

Demographic history of each *Leopardus* sample inferred with MSMC2 (Wang et al. 2020). The x-axis shows the coalescent time transformed to years, using a mutation rate $\mu = 8.6 \times 10^{-9}$ and a generation time of 3.8 years (Wang et al. 2022). The y-axis shows the effective population size ($N_e$). For each sample, dotted lines show 20 bootstrap replicates and the consensus of the replicates is shown as a bold line.

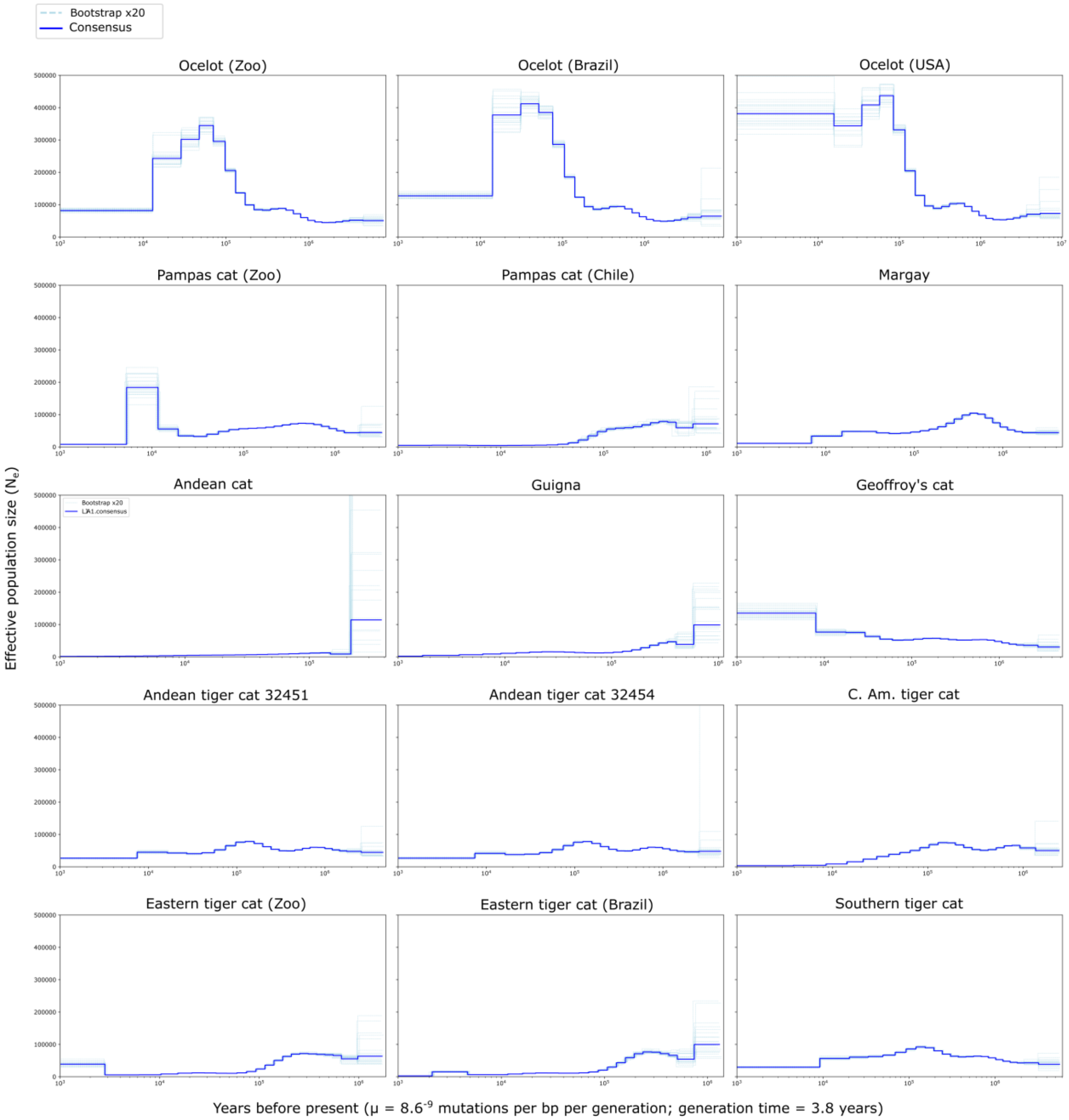

Supplement: msad255_Supplementary_Data [file msad255_supplementary_data.pdf]
